# Loss of the orphan nuclear receptor NR2F6 enhances CD8^+^ T-cell memory via IFN-γ

**DOI:** 10.1038/s41419-021-03470-9

**Published:** 2021-02-15

**Authors:** Bojana Jakic, William J. Olson, Kerstin Siegmund, Victoria Klepsch, Janine Kimpel, Verena Labi, Dietmar Zehn, Gottfried Baier, Natascha Hermann-Kleiter

**Affiliations:** 1 Translational Cell Genetics, Institute of Pharmacology and Genetics, Medical University of Innsbruck, Innsbruck, Austria; 2 Institute of Virology, Medical University of Innsbruck, Innsbruck, Austria; 3 Institute of Developmental Immunology, Biocenter, Medical University of Innsbruck, Innsbruck, Austria; 4 Division of Animal Physiology and Immunology, School of Life Sciences Weihenstephan, Technical University of Munich, Freising, Germany; 5 Present Address: Department of Immunology, Genetics and Pathology, Uppsala University, Uppsala, Sweden; 6 Present Address: Institute for Biomedical Aging Research, University Innsbruck, Innsbruck, Austria

**Keywords:** Interferons, Bacterial infection

## Abstract

Memory formation is a hallmark of T cell-mediated immunity, but how differentiation into either short-lived effector cells (SLECs, CD127^−^KLRG1^+^) or memory precursors cells (MPECs, CD127^+^KLRG1^−^) and subsequent regulation of long-term memory is adjusted is incompletely understood. Here, we show that loss of the nuclear orphan receptor NR2F6 in germ-line *Nr2f6*-deficient mice enhances antigen-specific CD8^+^ memory formation up to 70 days after bacterial infection with *Listeria monocytogenes* (LmOVA) and boosts inflammatory IFN-γ, TNFα, and IL-2 cytokine recall responses. Adoptive transfer experiments using *Nr2f6*^*−/−*^ OT-I T-cells showed that the augmented memory formation is CD8^+^ T-cell intrinsic. Although the relative difference between the *Nr2f6*^*+/+*^ and *Nr2f6*^*−/−*^ OT-I memory compartment declines over time, *Nr2f6*-deficient OT-I memory T cells mount significantly enhanced IFN-γ responses upon reinfection with increased clonal expansion and improved host antigen-specific CD8^+^ T-cell responses. Following a secondary adoptive transfer into naïve congenic mice, *Nr2f6*-deficient OT-I memory T cells are superior in clearing LmOVA infection. Finally, we show that the commitment to enhanced memory within *Nr2f6*-deficient OT-I T cells is established in the early phases of the antibacterial immune response and is IFN-γ mediated. IFN-γ blocking normalized MPEC formation of *Nr2f6*-deficient OT-I T cells. Thus, deletion or pharmacological inhibition of NR2F6 in antigen-specific CD8^+^ T cells may have therapeutic potential for enhancing early IFN-γ production and consequently the functionality of memory CD8^+^ T cells in vivo.

## Introduction

The generation of effector and memory CD8^+^ T cells is a requirement for the clearance of intracellular pathogens and subsequent long-term protection, and both CD8^+^ subsets are targets for anticancer immunotherapy and vaccination^1^. Upon pathogen clearance, most effector CD8^+^ T cells undergo apoptosis, but a small number survives and is maintained as memory CD8^+^ T-cell pool^2^.

Memory CD8^+^ T-cell precursors form early in the antibacterial effector phase and can be characterized by effector functions, tissue localization, and cell surface molecules (such as CD44, CD62L, CD127, CD122, and CXCR3) (ref. ^3–12^). Notably, differential KLRG1 and CD127 expression distinguish CD8^+^ short-lived effector cells (SLECs, CD127^−^KLRG1^+^) from memory precursor effector cells (MPECs, CD127^+^KLRG1^−^)^2,9^. Furthermore, CXCR3 expression is essential for CD8^+^ T-cell priming, memory cell formation, and enhanced memory cell responses^5,8^. KLRG1 and CD127 appear causally linked to the expression of the T-box transcription factors T-bet and eomesodermin (Eomes), and relative levels of T-bet and Eomes determine effector (T-bet^hi^Eomes^lo^) or memory (T-bet^lo^Eomes^hi^) fate^9^. The asymmetric division of activated CD8^+^ T-cells can result in unequal inheritance of T-bet and supports the idea that fate decisions can occur prior to the MPEC/SLEC stage^2,9,10,13^.

Recently, roles for nuclear receptors (NR) in CD8^+^ effector and memory T-cell generation have been determined^14–18^. Using germ-line knockout mice, we have identified functions of the orphan NR, NR2F6 in effector CD4^+^ and CD8^+^ T-lymphocytes during cancer progression, autoimmune responses, and immunization^19–24^. Mechanistically, NR2F6 suppresses the transcription of cytokine genes directly (*Il21*) or via blocking the binding of activating transcription factors such as NFAT/AP-1 to the promoter and/or other conserved regulatory sequences (*Il2*, *Ifng*, *Il17*), thus acting as a brake on inflammatory cytokine responses^19,20,22,24^. Our previous tumor experiments demonstrated that *Nr2f6*-deficient mice have superior protective memory responses^19^. We, therefore, set out to further define the intrinsic role of NR2F6 in CD8^+^ T-cell memory responses.

## Material and methods

### Mice

CD45.1^+^ (*Ptprc*^*a*^), C57BL/6 (CD45.2^+^/*Ptprc*^*b*^), and OT-I mice were purchased from Charles River (Germany). OT-I mice were crossed onto an *Nr2f6*^−/−^ background^19^. All strains were bred in the house under specific-pathogen-free conditions. Both females and males older than 6 weeks were used, minimum 3 mice per group. Mice were age and sex-matched for individual experiments, in a non-randomized manner. Animal procedures were approved by the Austrian Federal Ministry of Education, Science and Research (BMWFW-66.011/0064-WF/V/3b/2016; BMWFW-66.011/0112-WF/V/3b/2017).

### LmOVA infection

Recombinant *Listeria monocytogenes* expressing ovalbumin (LmOVA) were a gift from H. Shen, University of Pennsylvania, and handled as previously described^25^.

### Naïve cell enrichment and ACT

Naïve (CD62L^+^CD44^−^) OT-I T cells from OT-I *Nr2f6*^*+/+*^ or OT-I *Nr2f6*^*−/−*^ mice were negatively enriched using a magnetic cell isolation kit (Miltenyi, 130-096-543). Totally, 2 × 10^4^ (d7 or later harvest), 1 × 10^6^ (d3 harvest), or 3 × 10^6^ (24 h harvest) isolated T cells were transferred intravenously into recipient mice, which were then infected with LmOVA after 24 h. Mice were excluded if OT-I engraftment was inefficient, checked on d1 after transfer by bleeding the mice. Mice were sacrificed at the indicated days. For memory transfer experiments (>70 days), CD8^+^CD45.1^−^CD45.2^+^ OT-I T cells from pooled lymph nodes and spleens were fluorescence-activated cell sorting (FACS)-sorted on a BD FACSAria^™^ III (BD Biosciences, Germany). Doublets and dead 7AAD^+^ cells were excluded. Sorted cells were transferred into naïve recipients, which were infected with 2 × 10^5^ colony forming units (CFU) LmOVA 4 h later, and sacrificed after 3 days or bled over time (d3, 7, and 14).

### In vivo IFN-γ blocking

Naïve OT-I T cells were enriched and transferred into congenic recipients as described above. The next day, mice were infected with 1 × 10^4^ CFU LmOVA. Within the first 24 h after infection, 75 µg of either anti-IFN-γ blocking antibody (Biolegend, Clone XMG1.2) or isotype control rat IgG (BioXCell, Clone 2A3) was injected intraperitoneally per mouse. Mice were sacrificed 7 days after infection or monitored up to the indicated number of days and then sacrificed.

### Flow cytometry

Cell suspension preparation: spleen and lymph nodes were harvested and mashed through a 100 μm cell strainer in IMDM (Sigma-Aldrich, I13390) supplemented with 10% FCS (Biowest, S1810-500), 1% l-Glutamine (Merck Millipore, K0282), and 1% Penicillin/Streptomycin (Sigma-Aldrich, A2213). Blood was collected either by cardiac puncture at the time of sacrifice or through the mandibular vein. Red blood cells from all organs and blood were lysed in erythrocyte lysis buffer as described previously^19^.

*Antibody staining*: Cell suspensions were incubated with antibodies at 4 °C for 30 min in 2% fetal calf serum (FCS)/phosphate-buffered saline, a complete list of antibodies used is within the supplemental information. Staining of SIINFEKL-specific CD8^+^ T cells using a PE-conjugated OVA-H-2K^b^ tetramer (Baylor College of Medicine, USA) was performed at room temperature, as per manufacturer’s instructions. For cytokine staining, cells were fixed with intracellular fixation buffer (Biolegend, 420801), followed by permeabilization and intracellular cytokine staining using permeabilization wash buffer according to manufacturer instructions (Biolegend, 421002). For intracellular transcription factors, Ki67 or Bcl-2 staining, cells were fixed and permeabilized using the FoxP3 staining buffer kit (eBioscience, 00-5523), as described by the manufacturer. For live/dead discrimination with 7AAD (Biolegend, 420403) and Annexin V PE (Biolegend, 640947), cells were washed and stained in HBSS supplemented with Mg^2+^ and 2% FCS. 7AAD was added to the stained cell suspension mix directly before the acquisition. Cells were measured on a BD FACSVerse^™^, BD FACSCanto^™^ II, or a BD LSRFortessa^™^ (all BD Biosciences, Germany) flow cytometer.

### Cytotoxicity measurement

For the detection of degranulation, cells were stimulated for 4 h with PBDu and ionomycin in the presence of an anti-CD107a-BV421 (Biolegend, clone 1D4B, 121617) antibody. Next, cells were stained with anti-CD107a-PE-Cy7 (Biolegend, clone 1D4B, 121619) and other surface antibodies, followed by intracellular granzyme B and perforin staining, by using the intracellular fixation buffer (Biolegend, 420801) and permeabilization buffer (Biolegend, 421002) as per manufacturer instructions.

### Cell stimulation ex vivo

Single-cell suspensions of splenocytes from infected *Nr2f6*^*+/+*^ or *Nr2f6*^*−/−*^ mice or mice that received adoptively transferred OT-I T cells were stimulated in vitro with peptide. Totally, 2 × 10^6^ cells were stimulated for 4 h with 1 mM SIINFEKL N4 peptide (OVA_257–264_, AnaSpec, USA) in the presence of Brefeldin A (Golgi plug, BD Biosciences 555029). Alternatively, the cells were stimulated with 50 ng/ml phorbol 12,13-dibutyrate (PBDu) (Sigma-Aldrich, P1269) and 500 ng/ml ionomycin (Sigma-Aldrich, I0634) in the presence of Brefeldin A for 4 h. The cells were then stained as described above. For detecting CXCL9 (Biolegend, clone MIG-2F5.5), cells were stimulated with recombinant mouse IFN-γ (Biolegend, 575304) and recombinant mouse TNF-α (Invitrogen, RMTNFAI) at 10 and 20 ng/ml, respectively, for 2 h, thereafter for 20 h with 1 µg/ml LPS (Sigma-Aldrich, L4391). In the final 4 h of stimulation, Brefeldin A was added before intracellular staining was performed as described above.

### RNA isolation and real-time PCR

Total RNA was isolated using the RNeasy^®^ Mini Kit (Qiagen). First-strand cDNA synthesis was performed using oligo (dT) primers (Promega) with the Qiagen Omniscript RT kit, as described previously^19^. Expression analysis was performed using real-time PCR with an ABI PRIM 7000 or ABI PRIM 7500fast Sequence Detection System with TaqMan gene expression assays (Applied Biosystems; m*Ifng*: Mm01168134_m1; m*Tbx21*: Mm00450960_m1); all expression levels were normalized to *Gapdh* (Applied Biosystems; Mm99999915_g1).

### Statistical analysis

Statistical analysis was performed using Prism 8.0. Unless otherwise indicated, experiments were repeated at least two times using a minimum of 3 mice per group. The normality of our data was evaluated by the Shapiro–Wilk test. When normally distributed, we performed statistical analysis with unpaired Student’s *t* test for samples with equal variance (*F* test), two-way ANOVA, or mixed-effects model (REML). If data were not normally distributed, a Mann–Whitney *U* test was used. Differences between means were investigated by Student’s *t* test, one-way ANOVA, or REML to calculate significance. A *p* value < 0.05 was considered statistically significant. *p < 0.05; **p < 0.01, ***p < 0.001. Results are shown as mean ± SD. Randomization, blinding, or sample size estimation tests were not applied for our animal studies.

## Results

### Loss of NR2F6 in mice results in increased CD8^+^ memory T-cell formation and enhanced antigen-specific recall cytokine responses up to 70 days after LmOVA infection

**Fig. 1:**
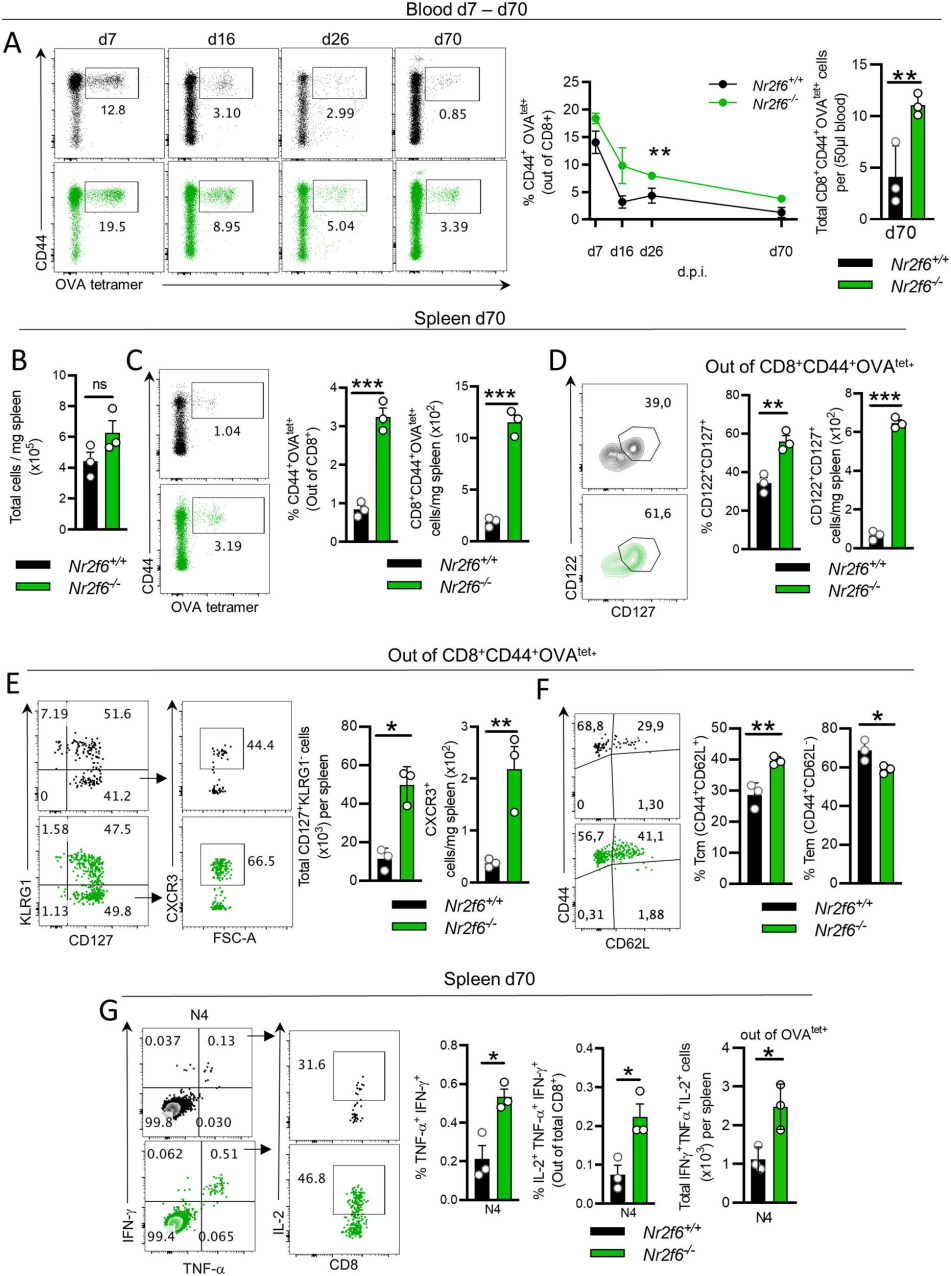
Loss of NR2F6 fosters the formation of antigen-specific CD8^+^ memory T cells and promotes an enhanced antigen recall effector cytokine response after LmOVA infection. **A** *Nr2f6*^*+/+*^ or *Nr2f6*^*−/−*^ mice were infected with 1 × 10^4^ CFU LmOVA. The frequency of antigen-specific (CD44^+^OVA^tet+^) cells was monitored in the blood for up to 70 d.p.i. Representative plots of CD44^+^OVA^tet+^ gated out of CD8^+^ T cells (left and middle) are shown on days indicated and total cell numbers per 50 μl of blood on day 70 (right). **B** *Nr2f6*^*+/+*^ and *Nr2f6*^*−/−*^ total number of cells (×10^5^) per mg of spleen, 70 d.p.i. **C** Representative plots and quantification of CD44^+^OVA^tet+^ specific CD8^+^ T cells in the spleen on 70 d.p.i. and the total number of CD44^+^OVA^tet+^ specific CD8^+^ T cells (×10^2^) per mg spleen. **D** Representative plots and quantification of CD127 and CD122 expression within CD8^+^CD44^+^OVA^tet+^ cells and numbers of memory CD8^+^CD44^+^OVA^tet+^CD122^+^CD127^+^ cells (×10^2^) per mg spleen, 70 d.p.i. **E** Representative plots and quantification of KLRG1 and CD127, and CXCR3 within CD127^+^KLRG1^−^ cells, all gated out of CD8^+^CD44^+^OVA^tet+^ cells and total numbers of CXCR3^+^ cells from the previous gates, shown as cells per mg of the spleen (×10^2^). **F** Representative plots and quantification of T central memory (Tcm) CD44^+^CD62L^+^ and T effector memory (Tem) CD44^+^CD62L^−^ expression gated out of CD8^+^CD44^+^OVA^tet+^ cells. **G** Representative dot plots depicting cytokine production within splenic CD8^+^ T cells d70 after LmOVA infection with N4 recall ex vivo for 4 h, in the presence of Brefeldin A. Graphs show quantification of *Nr2f6*^*+/+*^ or *Nr2f6*^*−/−*^ CD8^+^ T cells cytokine production as %, as total IFN-γ^+^TNF-α^+^IL-2^+^ producing cells per spleen (total cell numbers). Representative data shown are from one independent, out of at least two replicative experiments, with *n* = 3 per group and experiment. Each dot represents the data of an individual mouse. Results are shown as mean ± SD. An asterisk indicates statistically significant differences between genotypes calculated using Student’s *t* test or two-way ANOVA (A). A *p* value < 0.05 was considered statistically significant. *p < 0.05; **p < 0.01; ***p < 0.001; ns = not significant; d.p.i.: days post-infection.

To study the role of NR2F6 in memory CD8^+^ T cells, we infected germ-line *Nr2f6*^*−/−*^ or *Nr2f6*^*+/+*^ mice with LmOVA. Analyses over time revealed a significantly increased fraction of CD8^+^CD44^+^OVA^tet+^ T cells in the blood of *Nr2f6*-deficient mice (Fig. 1A). On d70 post infection, despite unaltered total cell counts in the spleen, we detected a relative as many as a threefold increase in *Nr2f6*-deficient CD8^+^CD44^+^OVA^tet+^ cells (Figs. 1B, C, and S1A). A higher frequency of CD8^+^OVA^tet+^ cells expressed the memory markers CD127 (IL-7R) and CD122 (IL-15Rβ) in *Nr2f6*-deficient mice (Fig. 1D). Finally, relative to *Nr2f6*-sufficient cells, the fraction of *Nr2f6*^−/−^ CD127^+^KLRG1^−^ cells expressing CXCR3 was significantly increased (Fig. 1E). Importantly, we detected a skewing of *Nr2f6*-deficient memory CD8^+^ cells toward central memory (Tcm, CD44^+^CD62L^+^) and away from effector memory cells (Tem, CD44^+^CD62L^−^) (Figs. 1F and S1A).

Of note, *Nr2f6*-deficiency did not impact pathogen clearance 3 days post-LmOVA infection (Fig. S1B). However, whereas the initial animal weight loss in both genotypes was comparable, *Nr2f6*-deficient mice had regained weight faster than wild-type controls on day 7 after infection (Fig. S1B). These results indicate that CD8^+^OVA^tet+^ persistence is not due to a failure to clear LmOVA. The fraction of cells carrying the exhaustion markers Lag-3, Tim-3, or PD-1 was comparable between *Nr2f6*^*+/+*^ and *Nr2f*6^−/−^ CD8^+^OVA^tet+^ T cells, indicating that *Nr2f*6^−/−^ cells do not become excessively exhausted (Fig. S1C)^26^, which is in contrasting our previous observations in tumor-infiltrating T cells^19^.

To investigate the effector functions of memory *Nr2f6*-deficient CD8^+^ T cells, we stimulated splenocytes ex vivo on d70 post infection either in an antigen-independent manner using phorbol 12,13-dibutyrate (PDBu) and ionomycin (P/I) or in an antigen-specific manner with the SIINFEKL (N4) peptide. We observed a significant increase in IFN-γ^+^TNF-α^+^IL-2^+^ triple producing (TP) *Nr2f6*^*−/−*^ CD8^+^ T cells in response to N4 peptide stimulation (Figs. 1G and S1D).

To exclude potential differences in CD8^+^ T-cell development, we investigated thymic subset distribution and thymocyte CD5 expression at steady-state and found no differences between *Nr2f6*^*+/+*^ and *Nr2f6*^*−/−*^ mice (Fig. S1E)^27^. Moreover, the immune cell composition in the naive periphery was also unaltered (Fig. S1F).

Altogether, germ-line loss of *Nr2f6* augments the formation of antigen-specific CD8^+^ memory T cells in vivo, which persist over time and raise an enhanced multifunctional cytokine response upon antigen-specific rechallenge ex vivo.

### Improved effector functions and enhanced memory precursor commitment in *Nr2f6*^*−/−*^ mice are CD8^+^ T-cell intrinsic

**Fig. 2:**
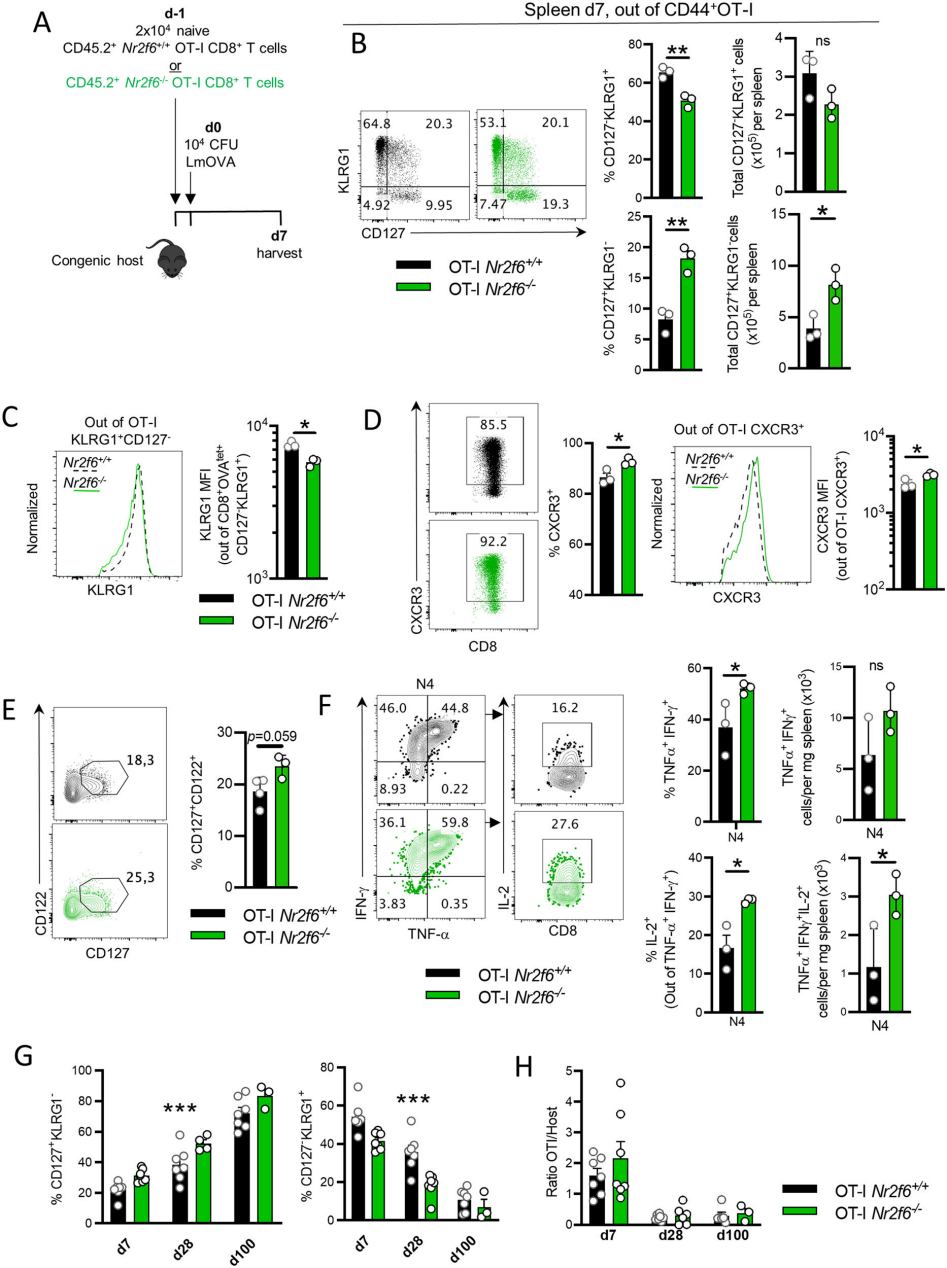
Enhanced memory formation in *Nr2f6*^*−/−*^ CD8^+^ T cells is cell-intrinsic. **A** Schematic overview of adoptive cell transfer of naïve CD45.2^+^ OT-I *Nr2f6*^*+/+*^ or *Nr2f6*^*−/−*^ *cells* into a congenic (CD45.1^+^ or CD45.1^+^CD45.2^+^) host, with LmOVA infection the day after and harvest on d7. One day after cell transfer the mice were infected with LmOVA. The analysis was performed 7 days post cell transfer. **B** Representative plots, quantification of frequencies and total cell numbers per spleen of SLECs (CD127^−^KLRG1^+^) and MPECs (CD127^+^KLRG1^−^) OT-I T cells at d7 after infection. Gated on CD45.2^+^CD45.1^−^CD8^+^CD44^+^. **C** Representative histogram of KLRG1 expression and MFI of KLRG1. **D** Representative dot plot and frequency of CXCR3 in *Nr2f6*^*+/+*^ or *Nr2f6*^*−/−*^ OT-I T cells. MFI out of CXCR3^+^ cells (positive cells from the dot plot) is shown, with a representative histogram. **E** Representative plots and frequency of CD122^+^CD127^+^ OT-I T cells. Gating is based on FMO controls. **F** Representative plots of cytokine production by transferred OT-I T-cells from spleen after recall ex vivo with N4 peptide, d7 post-LmOVA infection. **G** Frequencies of SLECs (CD127^−^KLRG1^+^) and MPECs (CD127^+^KLRG1^−^) OT-I T cells in the blood over time at indicated days after infection. **H** Ratio of OT-I over host CD8^+^ T cells in blood over time, at indicated days after infection. Representative data are shown from one independent, out of at least two replicative experiments, with *n* = 3 (**B**–**F**) per group and experiment. **G**, **H** Data are shown as pooled experiments of at least two independent experiments *n* = 3–7. Each dot represents the data of an individual mouse. Results are shown as mean ± SD. An asterisk indicates statistically significant differences between genotypes calculated using Student’s *t* test or mixed-effects model (REML) (**G**, **H**). A *p* value < 0.05 was considered statistically significant. *p < 0.05; **p < 0.01; ns not significant. All OT-I T cells are gated as CD45.2^+^CD45.1^−^CD8^+^CD44^+^.

To investigate whether the enhanced memory CD8^+^ T-cell formation in germ-line *Nr2f6*-deficient mice is CD8^+^ cell-intrinsic, we used an OT-I adoptive cell transfer (ACT), model. Totally, 2 × 10^4^ naïve CD45.2^+^OT-I T cells from *Nr2f6*^+/+^ or *Nr2f6*^−/−^ mice were transferred into separate naïve congenic recipients, which were then infected with LmOVA (Fig. 2A). Of note, surface expression of activation and memory markers on naïve OT-I T cells that were used for ACT were comparable (Fig. S2A).

We investigated MPEC and SLEC formation during the acute phase of the antibacterial response^2,9,10,13^. Similar to the *Nr2f6* germ-line knockout experiments, transferred *Nr2f6*^*−/−*^ OT-I T cells exhibited significantly more MPECs, both by cell number and frequency, and a decreased proportion of SLECs in the spleen (Fig. 2B). Although we did not observe changes in total cell numbers in the SLEC compartment, KLRG1 surface expression was significantly reduced within *Nr2f6*^*−/−*^ OT-I SLECs (Fig. 2C). Of note, MPEC and SLEC compartments from host CD8^+^CD44^+^OVA^tet+^ cells were similar irrespective of whether *Nr2f6*^*+/+*^ or *Nr2f6*^*−/−*^ transferred OT-I T cells were present (Fig. S2B). Within *Nr2f6*^*−/−*^ OT-I T cells, we observed an enhanced fraction of CXCR3^+^ cells and increased CXCR3 expression level, but not an increase in cell number (Figs. 2D and S2C). Although T-bet is reported to be a key driver of CXCR3 expression in CD8^+^ T cells^5,28^, we did not observe enhanced expression within the *Nr2f6*^*−/−*^ CXCR3^+^ population (Fig. S2D). Similar to d70 germ-line knockout mice, we observed a slight increase in CD127^+^ *Nr2f6*^*−/−*^ OT-I T cells co-expressing CD122 (Fig. 2E)^29^. Expression of other cytokine receptors of the γ-chain receptor family or exhaustion markers (Fig. S2E, F), proliferation or cell survival (Fig. S2G) were not altered between genotypes.

Upon restimulation with cognate antigen (N4) on day 7 ex vivo, we observed a significant increase in the fraction of *Nr2f6*^*−/−*^ OT-I T cells producing effector cytokines, in particular, triple producing (TP) (IFN-γ^+^TNF-α^+^IL-2^+^) was enhanced (Fig. 2F). To investigate if *Nr2f6*^*−/−*^ OT-I are binding larger amounts of the peptide and therefore exhibit enhanced TCR stimulation and cytokine production, we examined N4 peptide avidity in vitro^30^. We did not observe differences in proliferation, peptide-binding capacity, or CD3 downregulation, implying that the changes were seen in *Nr2f6*^*−/−*^ OT-I T cells are a result of intrinsic differences (Fig. S3A, B).

Finally, the d7 OT-I ACT results were recapitulated in germ-line *Nr2f6*-deficient mice, including a higher percentage of CD44^+^OVA^tet+^ cells in the peripheral blood and a similar trend in the spleen (Fig. S4A, B). Functional analysis of the CD8^+^OVA^tet+^ T cells resembled the OT-I adoptive transfer model’s phenotype with enhanced expression of IFN-γ and TNF-α, but not IL-2 (Fig. S4C). Moreover, we did not observe enhanced cytotoxicity (Fig. S1D).

The results from the ACT of *Nr2f6*^*−/−*^ OT-I T cells imply that loss of NR2F6 enhances antigen-specific memory precursor formation in vivo and effector cytokine production following acute LmOVA infection in a CD8^+^ T-cell intrinsic manner.

### Enhanced memory *Nr2f6*^−/−^ OT-I CD8^+^ T cells persist after the contraction phase but wane long term

To investigate the long-term persistence and phenotype of *Nr2f*6-deficient OT-I T cells, we tracked transferred cells in the blood over 100 days following primary infection (Fig. 2G). Enhanced frequencies of CD127^+^KLRG1^−^ *Nr2f6*^*−/−*^ OT-I cells persisted after the contraction phase till day 28, but subsequently, the difference in the blood faded and was not detectable on day 100 (Fig. 2G and Fig. S7A see below). We also observed a gradual loss of OT-I T cells (Fig. 2H). Despite the eventual decline in the blood, the enhanced memory phenotype of OT-I T cells in the spleen persisted until >d60 (Fig. 6D, see below), similar to the results with LmOVA infected germline-*Nr2f6*^*−/−*^ mice. In contrast, by d140 after infection, we could not observe any differences in splenic *Nr2f*6-deficient OT-I CD127^+^KLRG1^−^, Tcm, or Tem populations (Fig. S5A–C). Moreover, the number of *Nr2f*6-deficient OT-I T cells per spleen was equal between genotypes (Fig. S5D). As on d7 ACT, we did not observe any differences in host CD8^+^OVA^tet+^ populations by >d140 after infection (Fig. S5E, F).

Taken together, these results indicate that the phenotypical differences between *Nr2f6*^*+/+*^ and *Nr2f6*^*−/−*^ OT-I T cells persist after the contraction phase (days 60–70) but disappear long-term (>d140), in both the blood and the spleen.

### *Nr2f6*-deficient memory OT-I CD8^+^ T cells raise enhanced recall responses and are superior in clearing secondary bacterial infection

**Fig. 3:**
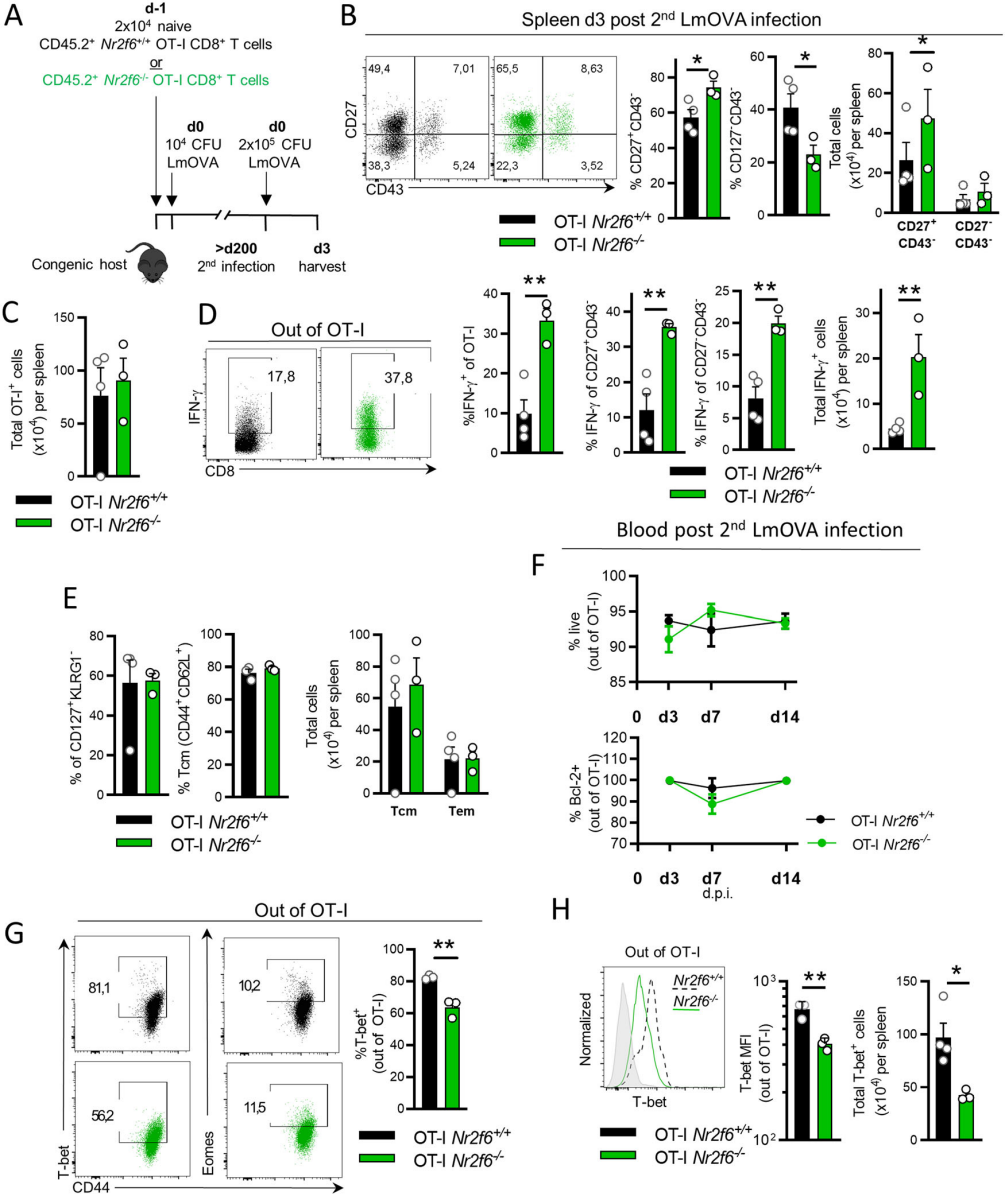
*Nr2f6*^*−/−*^ OT-I CD8^+^ memory T cells display enhanced effector cytokine responses upon secondary infection, despite no alterations in the memory phenotype. **A** Schematic overview of secondary infection. Congenic mice were reconstituted with either CD45.2^+^ OT-I *Nr2f6*^*+/+*^ or *Nr2f6*^*−/−*^ T-cells and infected with 10^4^ CFU LmOVA and were re-infected > 200 days with 2 × 10^5^ CFU LmOVA (d0). Spleens were harvested on d3 after second infection and analyzed. **B** Representative dot plots, frequencies, and total cell numbers per spleen of memory T-cell subsets based on differential surface expression of CD27 and CD43 gated out of transferred OT-I. **C** Total number of OT-I cells per spleen. **D** Representative dot plots of IFN-γ ^+^ production within splenic OT-I at day 3 post LmOVA re-infection after N4 recall ex vivo for 4 h, in the presence of Brefeldin A. Frequencies of IFN-γ within CD27^+^CD43^−^ and CD27^−^CD43^−^ memory populations, as well as total OT-I and IFN-γ producing cells per spleen. **E** Quantification of MPEC frequencies (CD127^+^KLRG1^−^), frequencies, and total cell numbers of central memory (Tcm) CD44^+^CD62L^+^ and effector memory (Tem) CD44^+^CD62L^−^ OT-I T cells. **F** Frequencies of live (7-AAD^-^AnnexinV^−^), and Bcl-2^+^ OT-I T cells in the blood over time at indicated days after second LmOVA infection. **G** Representative dot plots of transcription factors T-bet and Eomes, in *Nr2f6*^+/+^ or *Nr2f6*^−/−^ OT-I. Gating is based on FMO controls. Quantification of % T-bet^+^ *Nr2f6*^+/+^ or *Nr2f6*^−/−^ splenic OT-I is shown. **H** Representative histograms (T-bet) and quantification of T-bet MFI together with total cell numbers in *Nr2f6*^+/+^ or *Nr2f6*^−/−^ OT-I T cells. Representative data are shown from one independent, out of at least two replicative experiments, with *n* = 3–4 per group and experiment. Each dot represents the data of an individual mouse. Results are shown as mean ± SD. An asterisk indicates statistically significant differences between genotypes calculated using Student’s *t* test, mixed-effects model (REML) (**F**), or two-way ANOVA (**E**). A *p* value < 0.05 was considered statistically significant. *p < 0.05; **p < 0.01. All OT-I T cells are gated as CD45.2^+^CD45.1^−^CD8^+^CD44^+^OVA^tet+^.

As immunological memory is characterized by the ability to mount recall responses with higher magnitude, we investigated the functional properties of *Nr2f6*-deficient OT-I memory T cells upon antigen re-encounter in vivo^31^. As we did not observe differences in the transferred OT-I memory cell compartments between genotypes on d140 after infection, we re-infected the same host mice 200 days after the initial infection using a 10-fold higher LmOVA dose and analyzed CD8^+^ T-cell responses on d3 after the second infection (Fig. 3A). We compared the frequencies of the memory populations based on CD27 and CD43 expression. CD27^hi^CD43^lo^ cells have been shown to maintain long-term memory and demonstrate superior cellular expansion upon antigen re-encounter, whereas CD27^lo^CD43^lo^ cells display superior protective immunity against *Listeria* or *Vaccinia* virus re-infection, despite less expansion^31^. Upon re-infection, we detected an enhanced frequency of *Nr2f6*-deficient OT-I T cells CD27^+^CD43^−^ cells and lower levels of CD27^−^CD43^−^, but no difference in cell numbers between genotypes of the latter (Fig. 3B, C).

Upon restimulation with N4 ex vivo, memory *Nr2f6*^*−/−*^ OT-I T cells produced significantly more IFN-γ, but not TNF-α when compared to *Nr2f6*^*+/+*^ OT-I T cells (Fig. 3D and data not shown). Importantly, IFN-γ production was enhanced both in the CD27^+^CD43^−^ and CD27^−^CD43^−^ memory subsets (Fig. 3D). Despite unaltered total OT-I T cell numbers in the spleen, IFN-γ^+^ cell numbers were enhanced, suggesting a more robust IFN-γ response in *Nr2f6*^*−/−*^ OT-I T cells upon re-infection with the same pathogen (Fig. 3C, D). The frequency of CD127^+^KLRG1^−^ and the Tcm/Tem OT-I T cell compartments were unaltered between genotypes in the spleen (Fig. 3E), similar to late time-points before secondary infection (Fig. S5).

Of note, in the blood, neither the frequency nor total numbers of live or Bcl-2^+^ OT-I T cells were altered on day 3, 7, or 14 post second LmOVA infection (Fig. 3F and data not shown).

Interestingly, *Nr2f6*^*−/−*^ OT-I T cells contained a reduced fraction of T-bet^+^ cells and generally lower T-bet levels compared to *Nr2f6*^*+/+*^ OT-I T cells, but no difference in Eomes (data not shown) (Fig. 3G, H).

**Fig. 4:**
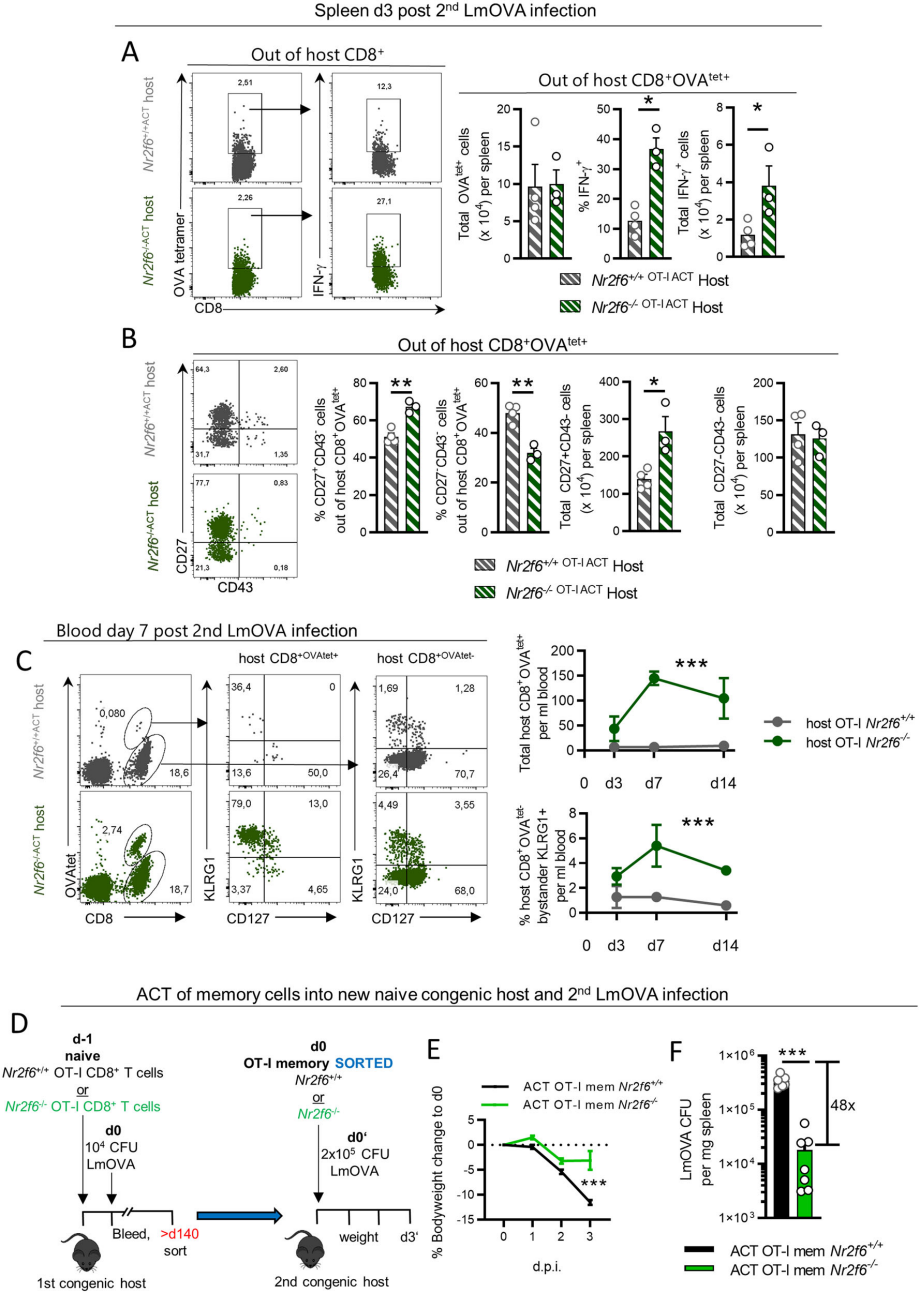
Host response after second LmOVA infection and ACT of OT-I CD8^+^ memory T cells with second LmOVA infection. **A** Representative dot plots, frequencies, and cell numbers of host CD8^+^OVA^tet+^ and IFN-γ production 3 days after second LmOVA infection, >d200 after the first infection. **B** Representative dot plots, frequencies, and cell numbers of host CD27^+^CD43^−^ and CD27^−^CD43^−^ memory populations 3 days after second LmOVA infection, >d200 after the first infection, gated out of CD8^+^OVA^tet+^. **C** Representative dot plots, frequencies, and cell numbers of host CD8^+^OVA^tet+^ and of host CD8^+^OVA^tet−^ cells together with SLECs (CD127^−^KLRG1^+^) and MPECs (CD127^+^KLRG1^−^) cells within both host CD8^+^OVA^tet^ populations are shown in the blood over time at indicated days after second LmOVA infection. **D** Schematic overview of the experimental setup of OT-I memory adoptive transfer and recall infection in vivo. Memory OT-I T cells were sorted >d140 after ACT and primary LmOVA infection and 5 × 10^4^ cells were transferred into naïve congenic hosts with subsequent high-dose (2 × 10^5^ CFU) LmOVA infection and harvest on day 3 after the second LmOVA infection. **E** Body weight of naïve congenic hosts transferred with memory *Nr2f6*^*+/+*^ or *Nr2f6*^*−/−*^ OT-I T cells compared to bodyweight at d0. **F** Quantification of CFUs in the spleen on day 3 post recall LmOVA infection. Representative data in **A**, **B** from one individual, out of at least two replicative experiments, with 3–4 mice per group per experiment, is shown. Pooled data in **E**, **F** from two replicative memory transfer experiments, with 3–4 mice per group, per experiment. Each dot represents data of an individual mouse. Results are shown as mean ± SD. An asterisk indicates statistically significant differences between genotypes calculated using Student’s *t* test. A *p* value < 0.05 was considered statistically significant. *p < 0.05; **p < 0.01; ***p < 0.001.

Unexpectedly, host CD8^+^OVA^tet+^ T cells produced significantly more IFN-γ (Fig. 4A), and CD8^+^OVA^tet+^CD27^−^CD43^−^ host T cells were significantly increased in number in the presence of *Nr2f6*-deficient OT-I memory T cells (Fig. 4B). Enhanced total cell numbers of host antigen-specific CD8^+^ T cells could also be detected in the blood on day 7 and 14 post second LmOVA infection. Surprisingly, bystander OVA^tet^-negative CD8^+^ T cell responses were also enhanced in the blood between day 3 and 14 post second LmOVA infection (Fig. 4C). These results collectively suggest that *Nr2f6*-deficient OT-I T cells might prime host CD8^+^ T cells via a secreted factor and subsequently influence secondary host memory CD8^+^ responses.

In order to investigate the antibacterial properties of *Nr2f6*-deficient OT-I memory T cells independently from the increased host responses in vivo, we used a secondary adoptive transfer model. Total *Nr2f6*^*+/+*^ and *Nr2f6*-deficient OT-I T cells were sorted between d140 and d160 after the initial LmOVA infection (Fig. S6A) and were transferred into new naïve congenic recipients (Fig. 4D) and infected with a high-dose of LmOVA. Three days later, host mice that had received *Nr2f6*-deficient memory OT-I T cells raised superior protective immunity measured by lower weight loss and a 48-fold reduction in splenic CFUs (Fig. 4E, F).

Thus, despite no noticeable phenotypic differences in the *Nr2f6*-deficient long-term OT-I memory T cells (>d140), upon rechallenge in vivo, *Nr2f6*-deficient OT-I memory T cells raise enhanced effector responses influencing the host CD8^+^ T-cell responses and resulting in superior protective antibacterial recall immunity on a per-cell basis.

### *Nr2f6*^*−/−*^ OT-I CD8^+^ T cells secrete enhanced IFN-γ levels during the early immune response

**Fig. 5:**
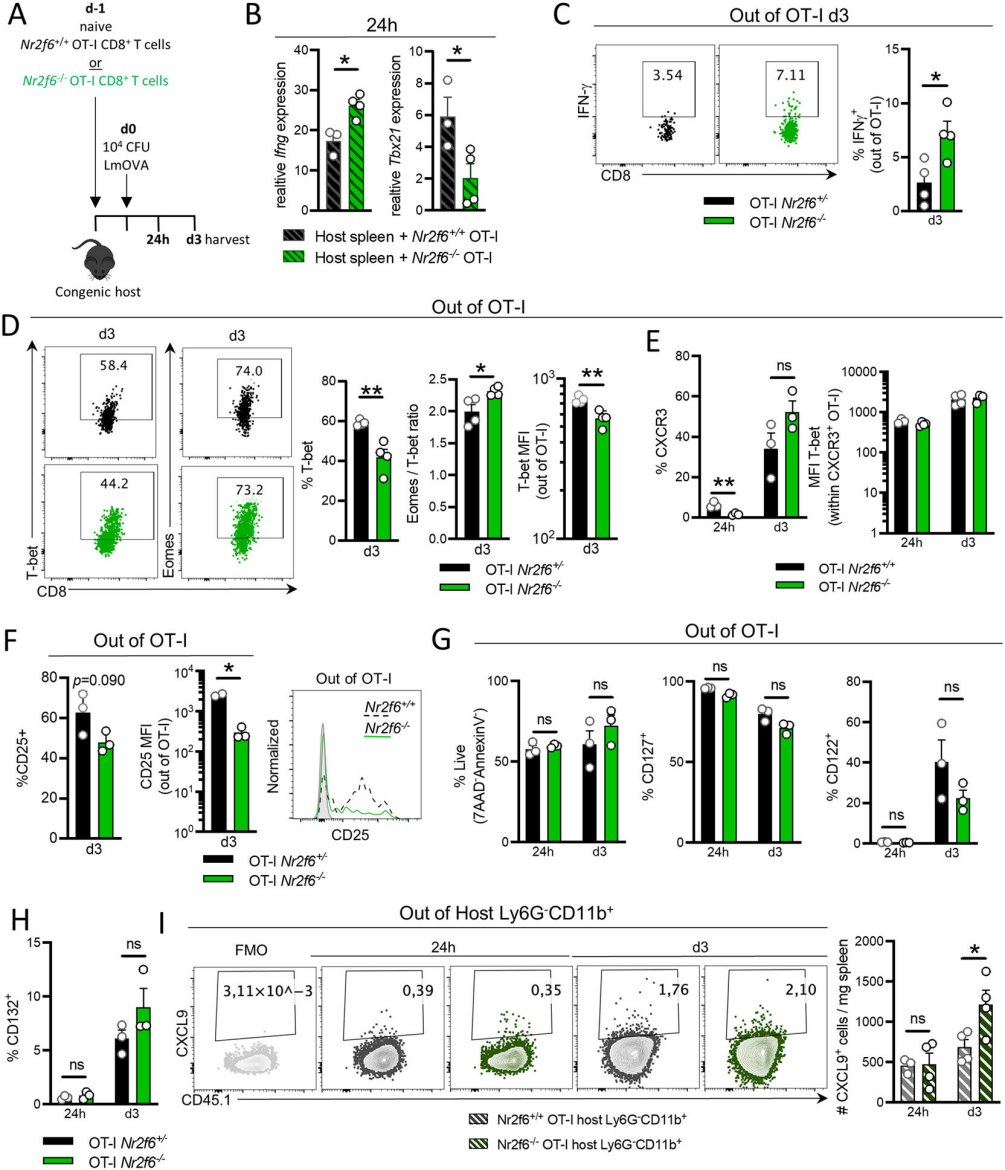
Loss of *Nr2f6* alters Eomes/T-bet ratio, enhances IFN-γ in *Nr2f6*^*−/−*^ OT-I CD8^+^ T cells during the early response to LmOVA, and induces CXCL9 production by host innate cells. **A** Totally, 1–3 × 10^6^ naïve *Nr2f6*^*+/+*^ or *Nr2f6*^*−/−*^ OT-I cells were transferred into congenic recipients. The next day, the mice were infected with LmOVA and sacrificed 24 h or 3 days after infection. Spleens were harvested and analyzed for CD8^+^ T cells responses. **B** Quantification of RT-PCR data of *Ifng* and *Tbx21* (T-bet) relative expression in the spleen of congenic recipients that received *Nr2f6*^*+/+*^ or *Nr2f6*^*−/−*^ OT-I cells, 24 h after infection. **C** Representative dot plots and frequency of *Nr2f6*^*+/+*^ and *Nr2f6*^*−/−*^ OT-I IFN-γ production on d3 after LmOVA infection. **D** Representative dot plots of T-bet and Eomes expression in OT-I. Frequency of T-bet^+^, Eomes/T-bet ratio, and T-bet MFI in OT-Is on d3 after LmOVA infection. **E** Frequency of CXCR3^+^ OT-I T cells, and MFI T-bet within CXCR3^+^ OT-I T-cells, 24 h and d3 after LmOVA infection. **F** Frequency of CD25^+^ OT-I T cells, a representative histogram of CD25 expression, and graph of CD25 MFI in OT-I cells, on d3 after infection. Gray histogram shows FMO. **G** Percentage of live cells (7AAD^−^AnnexinV^−^), CD127, CD122, and (**H**) CD132 in splenic OT-I T cells on d3 after infection. **I** CXCL9 production by (*Nr2f6*^*+/+*^) host splenic Ly6G^−^CD11b^+^ cells after IFN-γ and TNF-α priming for 4 h, followed by LPS stimulation ON, in the presence of transferred *Nr2f6*^*+/+*^ or *Nr2f6*^*−/−*^ OT-I T cells. Representative plots and quantification for CXCL9 by host Ly6G^−^CD11b^+^ cells per mg spleen are shown. Representative data are shown from one independent, out of at least two replicative experiments, with *n* = 3–4 per group and experiment. Each dot represents the data of an individual mouse. Results are shown as mean ± SD. An asterisk indicates statistically significant differences between genotypes calculated using Student’s *t* test. A *p* value < 0.05 was considered statistically significant. *p < 0.05; **p < 0.01; ns not significant. All OT-I T cells are gated as CD45.2^+^CD45.1^−^CD8^+^CD44^+^.

CD8^+^ T-cell fate is established early following antigen-dependent activation. Therefore, to determine the early and causal factor(s) that alter precursor and long-term memory formation in *Nr2f6*-deficient CD8^+^ T cells, we investigated OT-I T cell responses 24 h or 3 days after infection (Fig. 5A). Expression of *Ifng* in total splenocytes of host mice was enhanced with *Nr2f6*^*−/−*^ OT-I transfer, whereas *Tbx21* expression was reduced already at 24 h (Fig. 5B). IFN-γ cytokine responses during the early phase of infection were significantly increased in *Nr2f6*^*−/−*^ OT-I T cells on d3 (Fig. 5C).

Consistent with previous long-term memory data, the percentage of T-bet^+^ OT-I T cells was significantly reduced when NR2F6 was absent, resulting in an altered Eomes/T-bet ratio by d3 (Fig. 5D). In contrast to d7, we observed reduced frequencies of *Nr2f6*^*−/−*^ OT-I CXCR3^+^ cells along with unchanged T-bet expression within the CXCR3^+^ cells (Fig. 5E). However, a trend to a reduced percentage of CD25 expressing *Nr2f6*^*−/−*^ OT-I T cells was observed, and CD25 expression was significantly reduced on d3 (Fig. 5F).

Again, we did not observe differences in *Nr2f6*^*+/+*^ or *Nr2f6*^*−/−*^ OT-I T cell numbers (Fig. S6B), viability, proliferation, survival, cytolytic proteins, or the early activation marker CD69 (Fig. S6C–E). We did also not observe any difference between genotypes in CD127, CD122, or CD132 expression on transferred OT-I cells after 24 h or d3 (Fig. 5G, H).

IFN-γ is a critical driver of CXCL9 production by innate immune cells, which is a ligand for CXCR3^5^. To investigate if *Nr2f6*-deficient OT-I T-cells alter CXCL9 production in innate host cells via enhanced IFN-γ, we stimulated splenocytes in vitro with LPS, IFN-γ, and TNF-α, where TNF-α and LPS do not induce CXCL9, but were included to resemble in vivo infection^5,32^. We observed a significant increase in the fraction of CXCL9-producing innate host cells from mice that had received *Nr2f6*^*−/−*^ OT-I T cells, how these are influencing long-term memory capacity needs further investigations (Fig. 5I).

Taken together, during early anti-bacterial responses, *Nr2f6*-deficient OT-I T cells secrete enhanced IFN-γ levels, which result in enhanced production of the chemokine CXCL9 by host innate cells. Moreover, *Nr2f6*^*−/−*^ OT I cells show enhanced activation as reflected by the accelerated CD25 downregulation.

### Blocking IFN-γ in vivo early during the response to LmOVA resets OT-I CD8^+^ T-cell memory formation

**Fig. 6:**
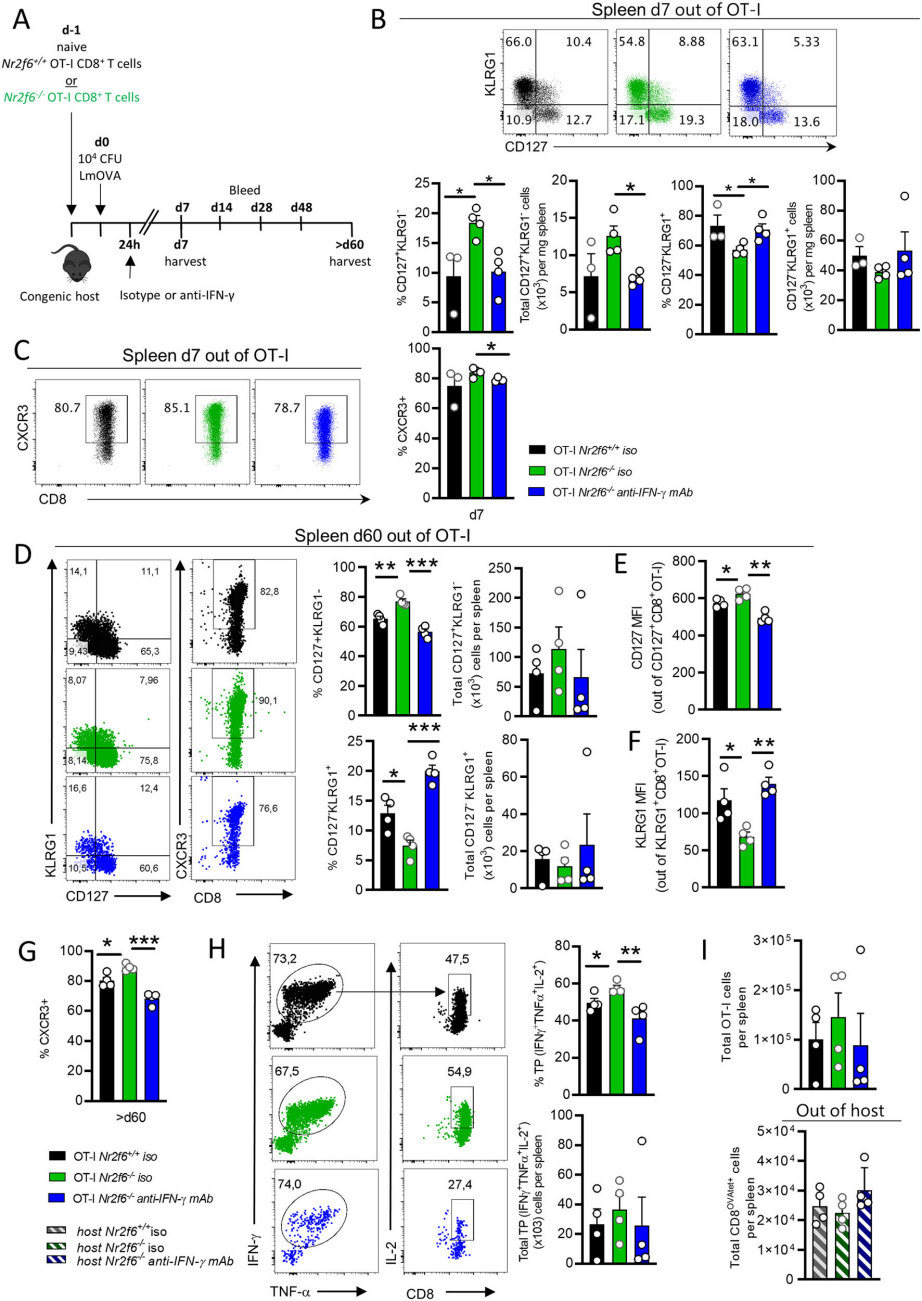
Blocking early IFN-γ resets the memory precursor phenotype in *Nr2f6*^*−/−*^ OT-I CD8^+^ T cells. **A** Scheme of the in vivo IFN-γ blocking strategy. Naive *Nr2f6*^*+/+*^ or *Nr2f6*^*−/−*^ OT-I T cells were transferred into the congenic host, which were infected 24 h later. Soluble IFN-γ was blocked 24 h after infection. Spleens were harvested 7 days or >d60 after infection and bled at the days indicated. **B** Representative dot plots, frequency and cell numbers of SLECs (CD127^−^KLRG1^+^) and MPECs (CD127^+^KLRG1^−^) OT-I on d7 after LmOVA infection and isotype control or IFN-γ blocking. **C** Representative dot plots and frequency of CXCR3^+^ cells on d7 *Nr2f6*^*+/+*^ or *Nr2f6*^*−/−*^ OT-I after LmOVA infection, after either isotype control or anti-IFN-γ treatment. **D** Representative dot plots, frequencies, and cell numbers of splenic SLECs (CD127^−^KLRG1^+^), MPECs (CD127^+^KLRG1^−^), and CXCR3^+^ cells on day 60 after either isotype control or anti-IFN-γ treatment. **E** Quantification of CD127 MFI. **F** Quantification of KLRG1 MFI. **G** Frequency of CXCR3^+^ on *Nr2f6*^*+/+*^ or *Nr2f6*^*−/−*^ OT-I from spleen on >d60 after infection, after either isotype control or anti-IFN-γ treatment. **H** Representative plots of cytokine production after recall ex vivo with N4 peptide, >d60 post LmOVA infection after either isotype control or anti-IFN-γ treatment. **I** Quantification of total transferred OT-I and host CD8^+^OVA^tet+^ T cells cell numbers in the spleen after either isotype control or anti-IFN-γ treatment. Representative data shown from one independent, with *n* = 3–4 per group and experiment. Each dot represents the data of an individual mouse. Results are shown as mean ± SD. An asterisk indicates statistically significant differences between genotypes calculated using Student’s *t* test. A *p* value < 0.05 was considered statistically significant. *p < 0.05; **p < 0.01; ns not significant. All OT-I T cells are gated as CD45.2^+^CD45.1^−^CD8^+^CD44^+^.

Recent work suggests that paracrine IFN-γ signaling as early as 24 h post infection with *Lm* is critical for OT-I T cell expansion, memory cell formation, and accelerates CD25 downregulation on antigen-specific CD8^+^ T cells^33^. To investigate whether enhanced IFN-γ production is a driver of increased memory formation of *Nr2f6*-deficient OT-I T cells, we injected an IFN-γ blocking antibody within 24 h after LmOVA infection (Fig. 6A).

Although we did not observe any change in CD119 (IFN-γR) expression in *Nr2f6*-deficient OT-I cells (Fig. S6F), by d7 after infection, SLECs had accumulated at the expense of MPECs in the spleens of anti-IFN-γ treated mice, resulting in similar levels as compared to animals that had received isotype control (Fig. 6B). In addition, the blockade of IFN-γ also leads to a reduction in CXCR3-expressing cells numbers (Fig. 6C).

The differences between isotype and IFN-γ blocked MPEC and SLEC *Nr2f6*-deficient OT-I T cells in blood persisted up to d28 after infection (Fig. S7A). Curiously, we observed a gradual loss of OT-I T cells in the animals that had received anti-IFN-γ, and these cells were nearly completely undetectable in the blood by d48 (Fig. S7A, B). The frequency of live cells (7AAD-AnnexinV−) was unaltered in the blood (Fig. S7C). Importantly, the differences between isotype and IFN-γ blocked CD127^−^KLRG1^+^ and CD127^+^KLRG1^−^ *Nr2f6*-deficient OT-I T cells in the spleen persisted up to >d60 after infection (Fig. 6D), similar to the d70 phenotype of germ-line *Nr2f6*-deficient mice (Fig. 1). Although the total cell numbers were not altered by >d60 after anti-IFN-γ treatment, cell surface expression of CD127 was significantly increased, whereas KLRG1 was significantly reduced (Fig. 6E, F). Furthermore, blockade of IFN-γ leads to a stable reduction of CXCR3 expressing *Nr2f6*-deficient OT-I T cells into the memory phase (>d60) (Fig. 6G).

**Fig. 7:**
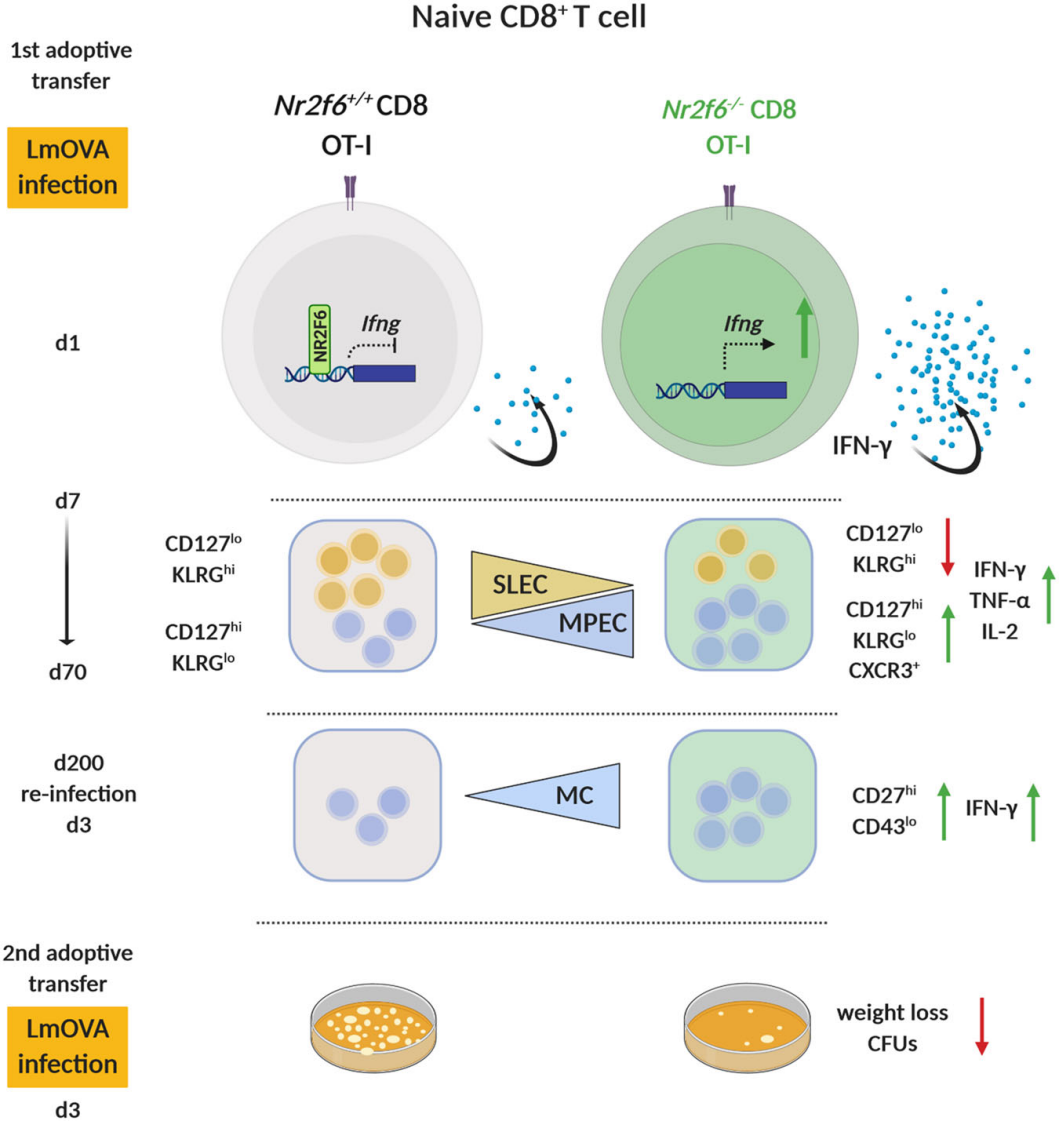
Loss of the orphan nuclear receptor NR2F6 enhances CD8^+^ T-cell memory via IFN. The nuclear orphan receptor NR2F6 limits *Ifng* transcription in the early phase of an anti-bacterial CD8^+^ T cell response (d1). Within an OT-I adoptive cell transfer model, intrinsic-loss of NR2F6 in CD8^+^ T cells augments IFN-γ secretion, which enhances the proportion of memory-precursor effector cells (MPECs CD127^hi^KLRG1h^lo^) over short-lived effector cells (SLECs CD127^lo^KLRG1^hi^). Furthermore, the secretion of the pro-inflammatory cytokines IFN-γ, TNF-α, and IL-2 are enhanced during the acute anti-bacterial response (d7). Enhanced memory *Nr2f6*^−/−^ OT-I CD8^+^ T-cells persist after the contraction phase (d70) but wane long-term (>d140). Upon re-infection (>d200), *Nr2f6*-deficient memory OT-I CD8^+^ T-cells raise enhanced recall responses and are – following adoptive transfer into naïve mice - superior in clearing secondary LmOVA infection.

Importantly, anti-IFN-γ block within *Nr2f6*-deficient OT-I cells reduced the frequency of IFN-γ^+^TNF-α^+^IL-2^+^ cells after restimulation with N4 peptide in vitro (Fig. 6H). Total transferred OT-I cells or host CD8^+^OVA^tet+^ T cells were not altered between genotypes or by anti-IFN-γ block (Fig. 6I). These results imply that IFN-γ positively affects CD8^+^ memory precursor cell commitment early during infection and is essential for memory CD8^+^ T-cell persistence and function. Suppression of IFN-γ production by NR2F6 during the initial phase of LmOVA infection subsequently controls effector vs. memory CD8^+^ T cell commitment, thus establishing NR2F6 as a central player in memory fate decision (Fig. 7).

## Discussion

CXCR3 expression, induced in a T-bet dependent manner in activated CD8^+^ T cells, is associated with enhanced migratory potential and effector function of CD8^+^ T cells^5^, optimal recall responses to *Listeria* infection and with the efficacy of anti-PD-1 tumor therapy^34,35^. However, the literature suggests that in *Cxcr3*^*−/−*^ mice memory CD8^+^ T cells accumulate, even after the viral infection has been cleared^6,36–38^. It was, therefore, surprising that *Nr2f6*-deficient CD8^+^ T cells expressed higher levels of CXCR3, despite reduced T-bet expression. NFATc1 has been shown to bind the CXCR3 promoter and control CXCR3 expression in CD8^+^ T cells^39^, independent of T-bet. We have previously shown that NFATc1 activity is enhanced in *Nr2f6*^*−/−*^ T cells. Thus, NFATc1 activity may be a driver of increased CXCR3 expression in *Nr2f6*-deficient CD8^+^ T cells^19^, especially as TF-binding analysis via TRANSFAC^®^ revealed several putative NFAT and NR2F (COUP) (TGACCT) DNA-binding sites within the murine CXCR3 promoter^40^. Furthermore, T-bet is considered a direct activator of IFN-γ transcription. However, in the context of CD8^+^ T cells, IFN-γ production has been shown to be independent of T-bet^28,41^, but dependent on Eomes^42^. In addition, T-bet drives the terminal differentiation of effector T cells while repressing self-renewal of memory CD8^+^ T cell^9,10,13^. We have previously shown that NR2F6 directly binds to the mouse *Ifng* promoter in CD8^+^ T cells and that loss of *Nr2f6* enhances IFN-γ expression, and secretion during the first 24 h after anti-CD3/CD28 stimulation in vitro. Our data with the LmOVA infection model show that lack of *Nr2f6* leads to increased IFN-γ expression, independently of T-bet^19,22^. Other NRs such as ERRα (NR3B1) or RARα (NR1F1) have been shown to regulate the *Tbx21* locus specifically in Tem but not in Tcm CD8^+^ T cells^43^. How and at which time point NR2F6 is initially involved in the regulation of the complex transcription factor network directing the induction of CD8^+^ memory T-cell fates requires future study.

Within the CD8^+^ T-cell pool, the production of pro-inflammatory cytokines such as IFN-γ or IL-2 is commonly associated with effector and cytotoxic cell subsets^44,45^. However, within the first 24 h of CD8^+^ T-cell activation, IFN-γ appears to prime for memory cell commitment^46,47^. Although IFN-γ can lead to enhanced memory formation via paracrine signaling or enhanced migration via autocrine signaling, we did not observe enhanced CD119 expression^33^. Instead, we suggest that the increase in host CXCL9, via IFN-γ, and CXCR3, via NFATc1, may result in optimal positioning of *Nr2f6*^*−/−*^ CD8^+^ T cells within the spatial organization in the spleen, and hence favor MPEC formation. By blocking IFN-γ at an early stage, before an apparent IFN-γ increase is observed, we show that indeed MPEC formation, as well as CXCR3 expression, is reduced. Interestingly, the loss of cells overtime after blocking IFN-γ indicates roles beyond MPEC formation that could possibly involve cell survival. How exactly CXCL9 influences T-cell activation and subsequent long-term memory in the context of NR2F6 needs further investigation.

We have previously shown that *Nr2f6* expression in CD8^+^ T cells increases following T-cell receptor (TCR) stimulation and acts as a transcriptional repressor that protects against excessive and potentially harmful cytokine secretion during the effector phase^19,21^, Interestingly, in a setting with lymphocytic choriomeningitis virus (LCMV), during the acute phase of the infection Tem expressed higher levels of *Nr2f6* when comparing to Tcm, whereas 180 days post infection the pattern was reversed (unpublished observations of the Immgen publicly available dataset). Although we did not investigate *Nr2f6* expression within SLECs and MPECs during the early and late phases of LmOVA infection, it does seem plausible that expression is higher in Tem during acute LCMV, i.e., NR2F6 is acting as a brake on cells with high potential to damage surrounding tissue. During the memory stage of LCMV, the higher expression in Tcm could serve a similar function, which is dampening nonspecific activation of memory cells. How and when Nr2f6 expression is changed in specific T-cell subsets during acute, memory, and recall stages of infection will need to be further elucidated in future studies.

The enhanced response by host CD8^+^ T cells after a second infection implies communication between OT-I cells and the host via one or more so far unidentified secreted factor(s). Although we mainly focused on IFN-γ in this context, recent literature has shown that CD28-CTLA4 signaling leads to a coordinated “all-or-none” response by CD8^+^ T cells that depends on population size and density, akin to the quorum sensing response observed in bacteria^46,47^. It is also worth noting that we did not observe enhanced host responses during the initial infection.

In summary, we show that loss of NR2F6 in CD8^+^ T cells leads to augmented antigen-specific memory formation and improved effector responses upon secondary infection that are mediated through an early burst of IFN-γ. The NR superfamily is a primary class of therapeutic drug targets for human disease, and dissecting the roles of NRs in CD8^+^ T cells is of importance^48^. Additional work needs to be done to evaluate precisely how IFN-γ in the context of NR2F6 regulates cell migration, intracellular signaling, and effector memory responses in infectious diseases and cancer.

## Supplementary information

Supplemantal Material Title page

Supplemantal Material Fig1

Supplemantal Material Fig2

Supplemantal Material Fig3

Supplemantal Material Fig4

Supplemantal Material Fig5

Supplemantal Material Fig6

Supplemantal Material Fig7
