# Supplementary figures and images for: Loss of the orphan nuclear receptor NR2F6 enhances CD8+ T-cell memory via IFN-γ
Source: Cell Death Dis. 2021 Feb 15;12(2):187. doi: 10.1038/s41419-021-03470-9 (PMC7884426; doi:10.1038/s41419-021-03470-9)

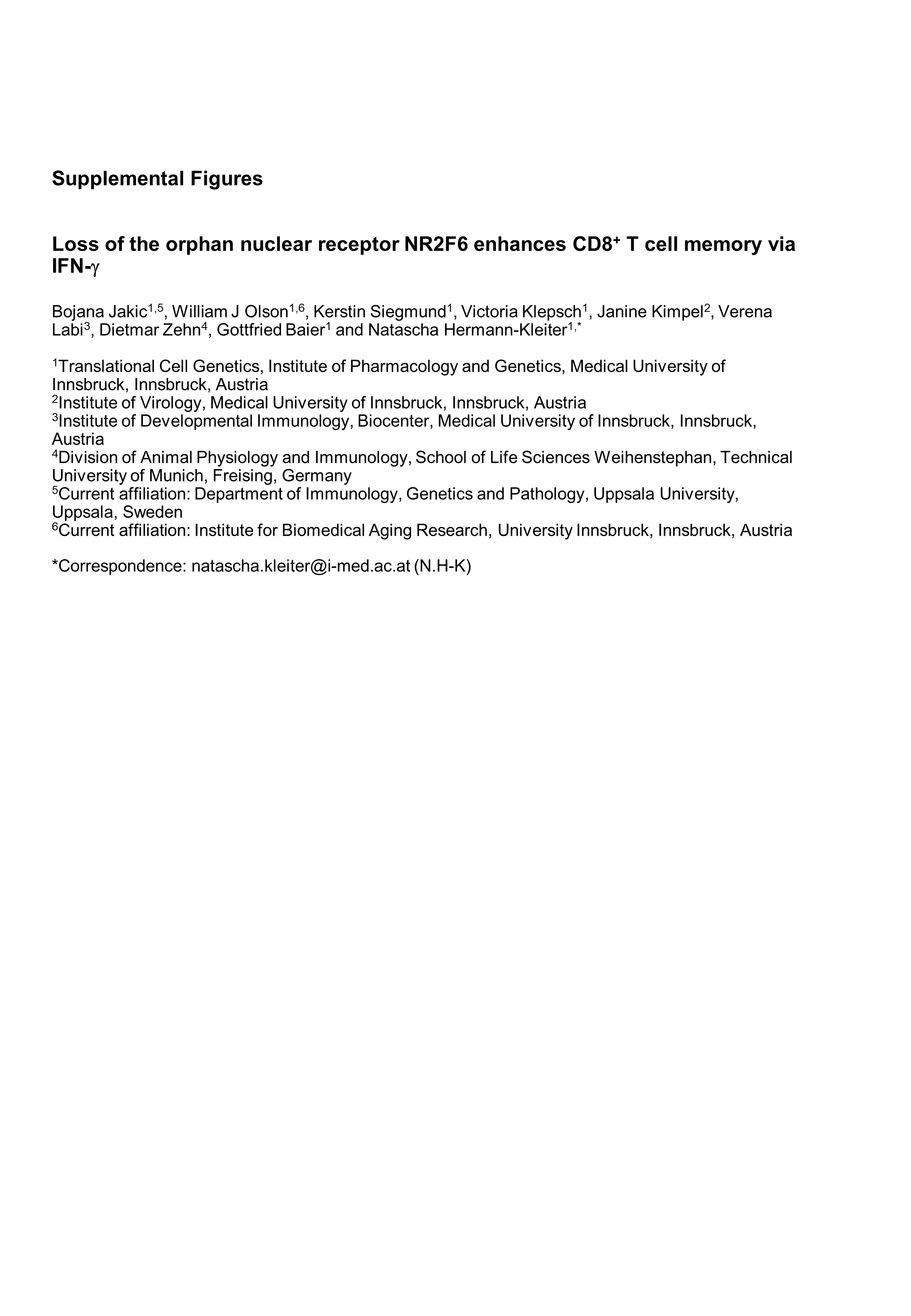

Supplement: Supplementary file 1 — Supplemantal Material Title page [file 41419_2021_3470_MOESM1_ESM.tif]

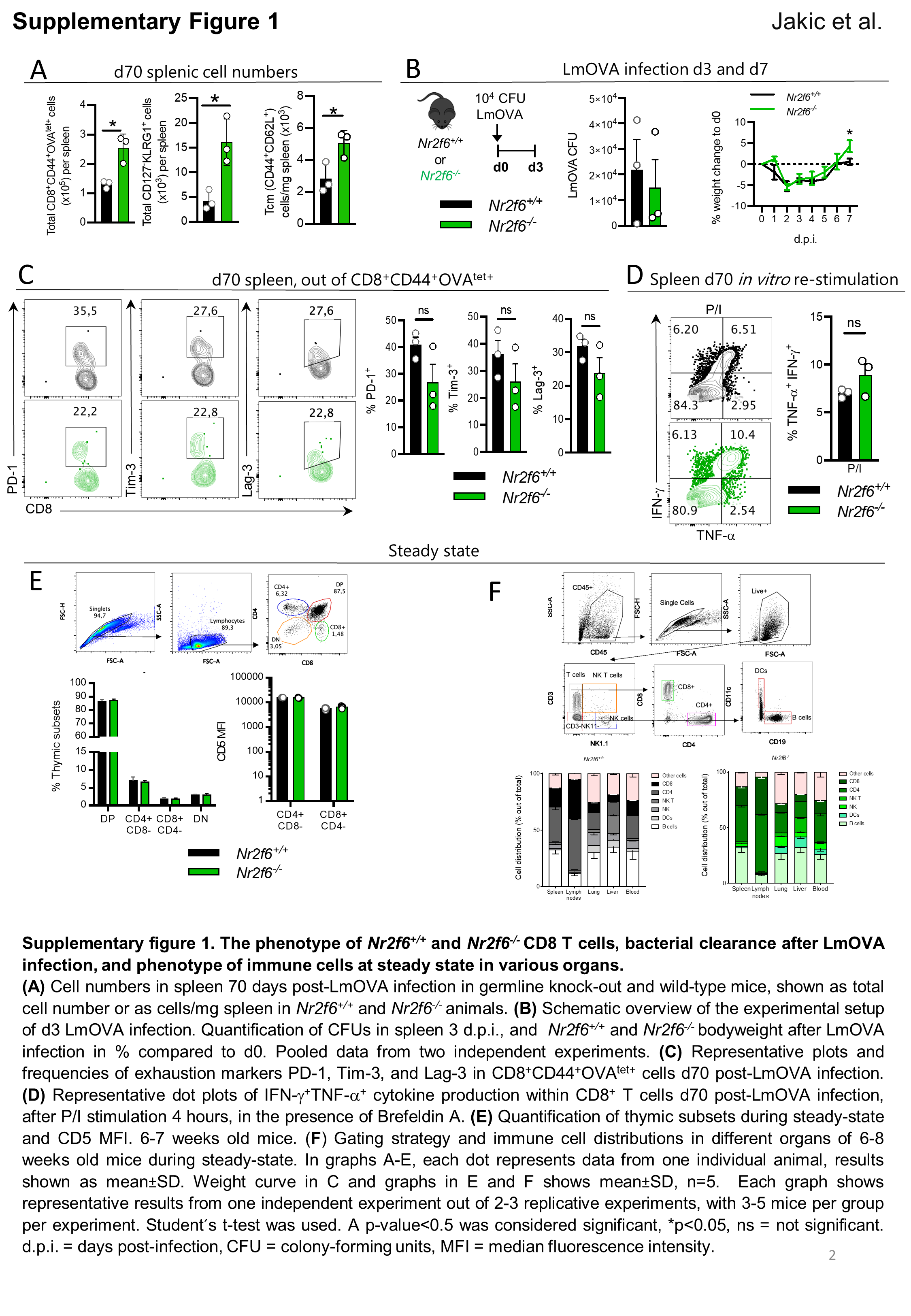

Supplement: Supplementary file 2 — Supplemantal Material Fig1 [file 41419_2021_3470_MOESM2_ESM.tif]

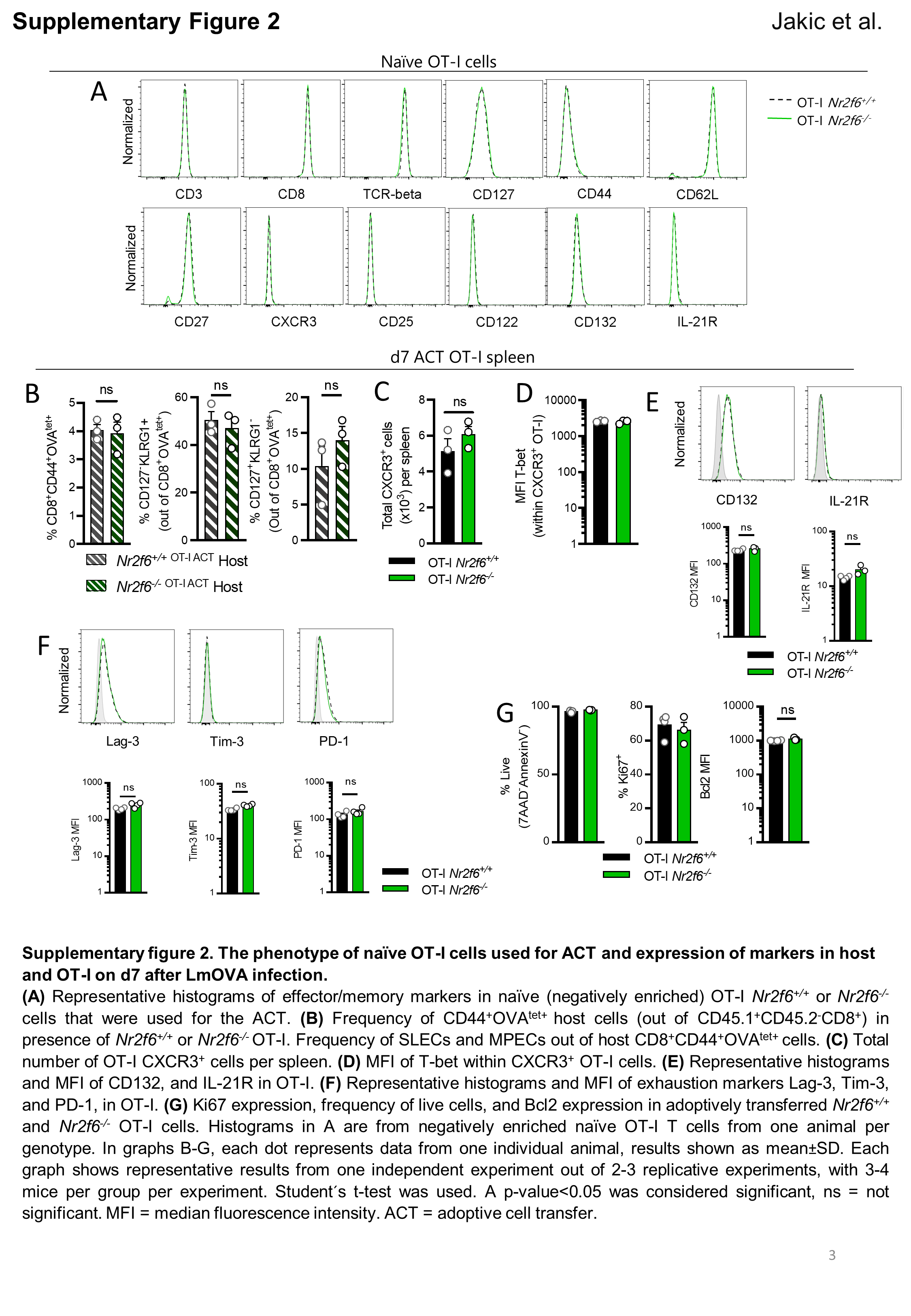

Supplement: Supplementary file 3 — Supplemantal Material Fig2 [file 41419_2021_3470_MOESM3_ESM.tif]

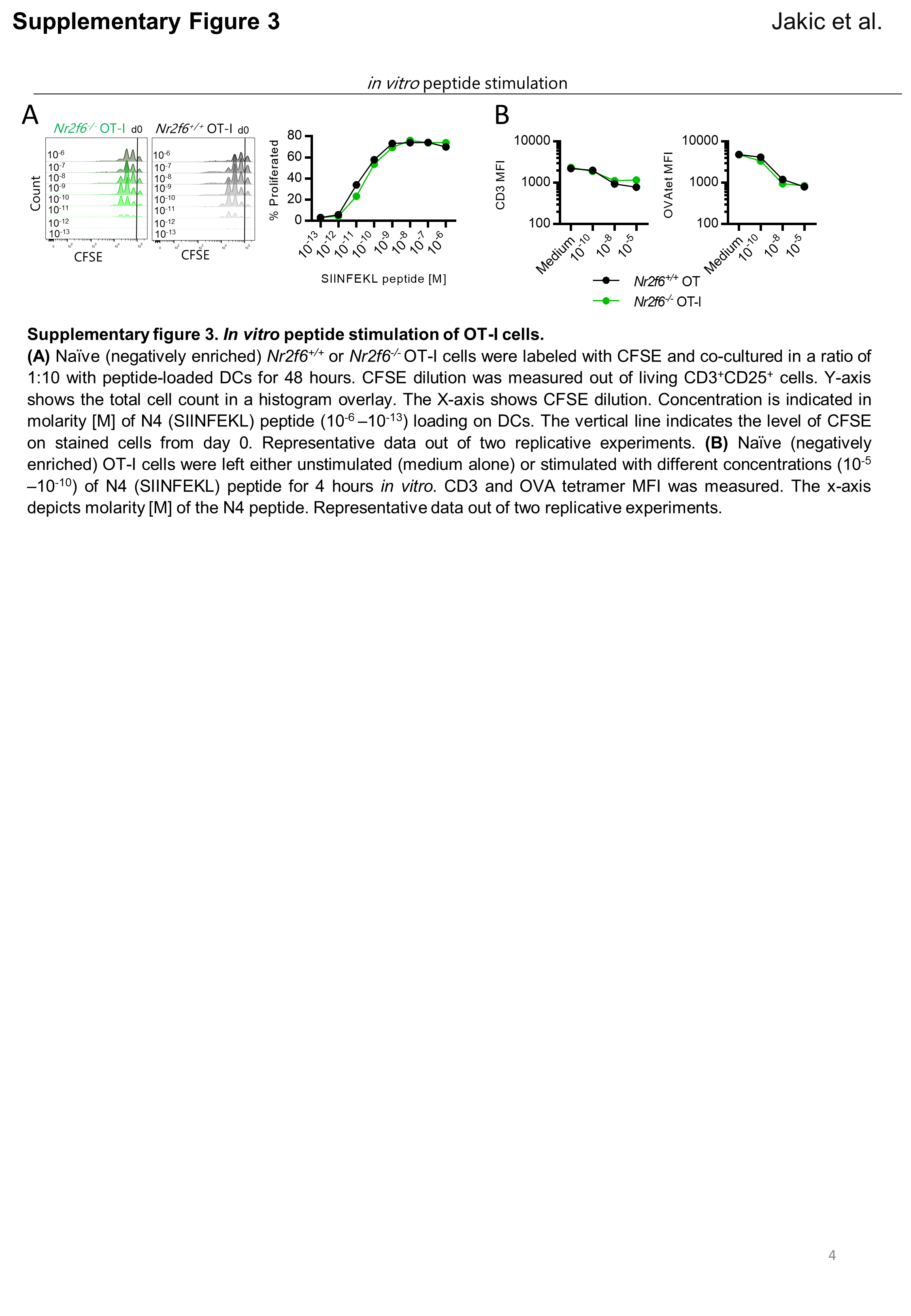

Supplement: Supplementary file 4 — Supplemantal Material Fig3 [file 41419_2021_3470_MOESM4_ESM.tif]

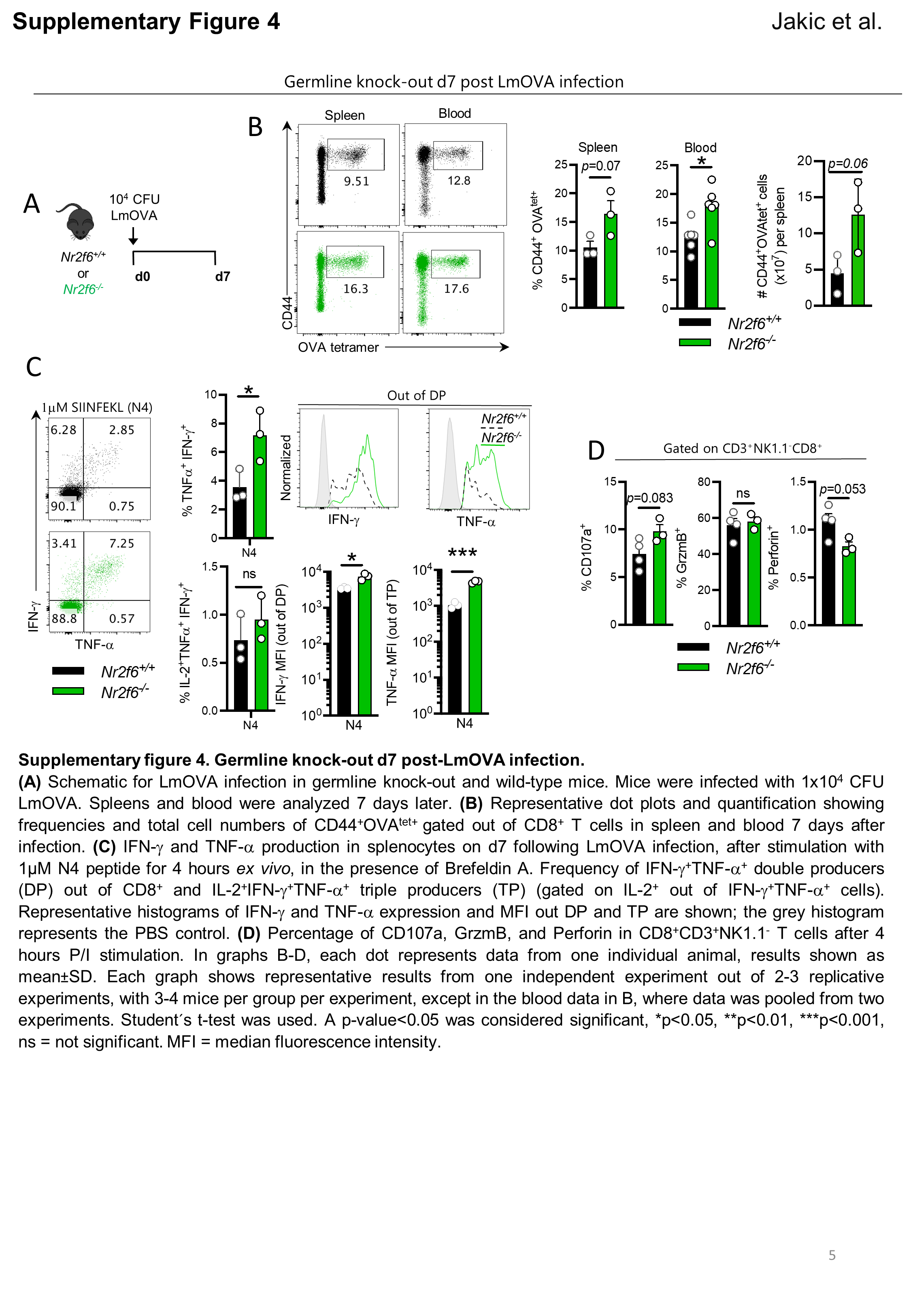

Supplement: Supplementary file 5 — Supplemantal Material Fig4 [file 41419_2021_3470_MOESM5_ESM.tif]

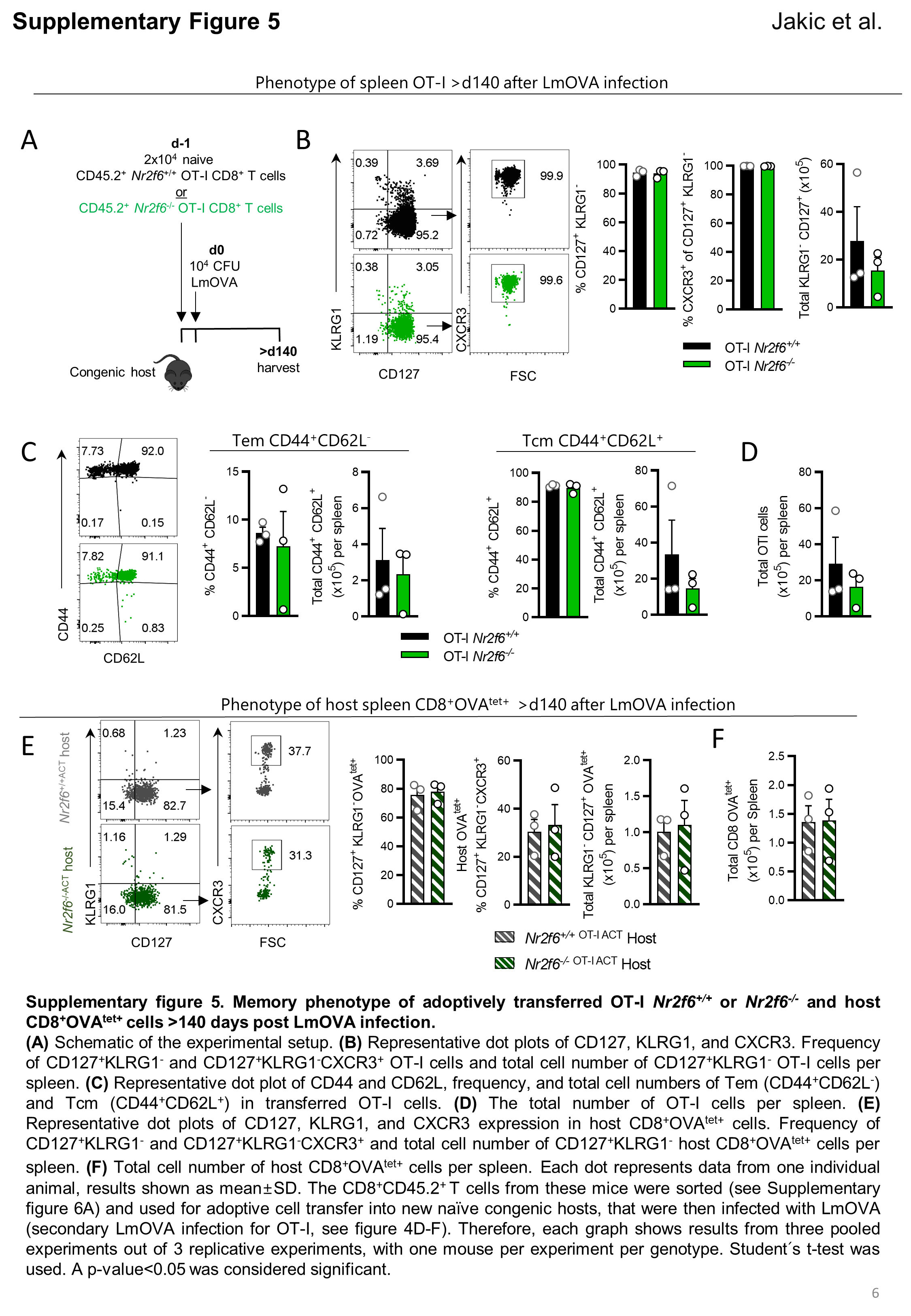

Supplement: Supplementary file 6 — Supplemantal Material Fig5 [file 41419_2021_3470_MOESM6_ESM.tif]

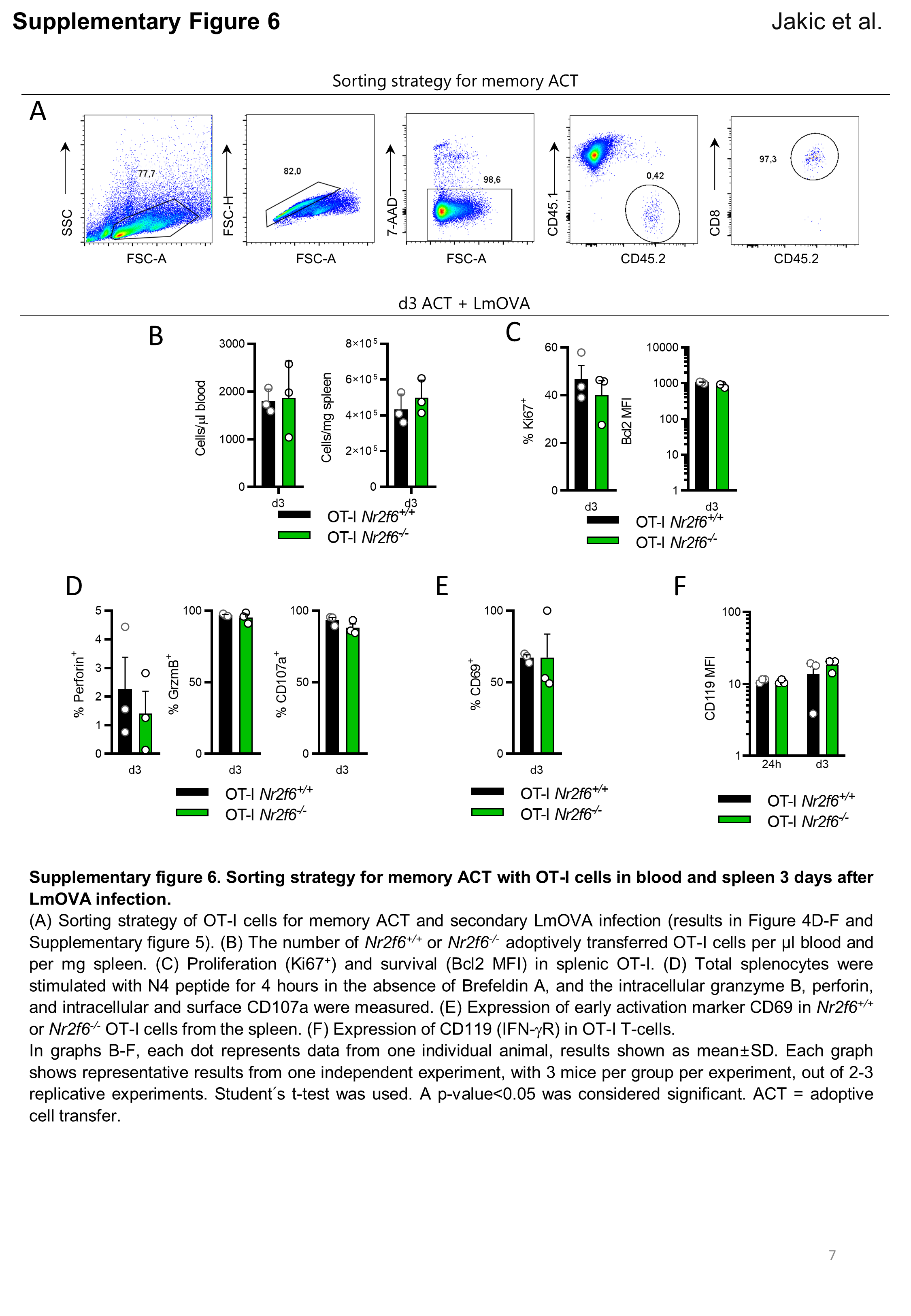

Supplement: Supplementary file 7 — Supplemantal Material Fig6 [file 41419_2021_3470_MOESM7_ESM.tif]

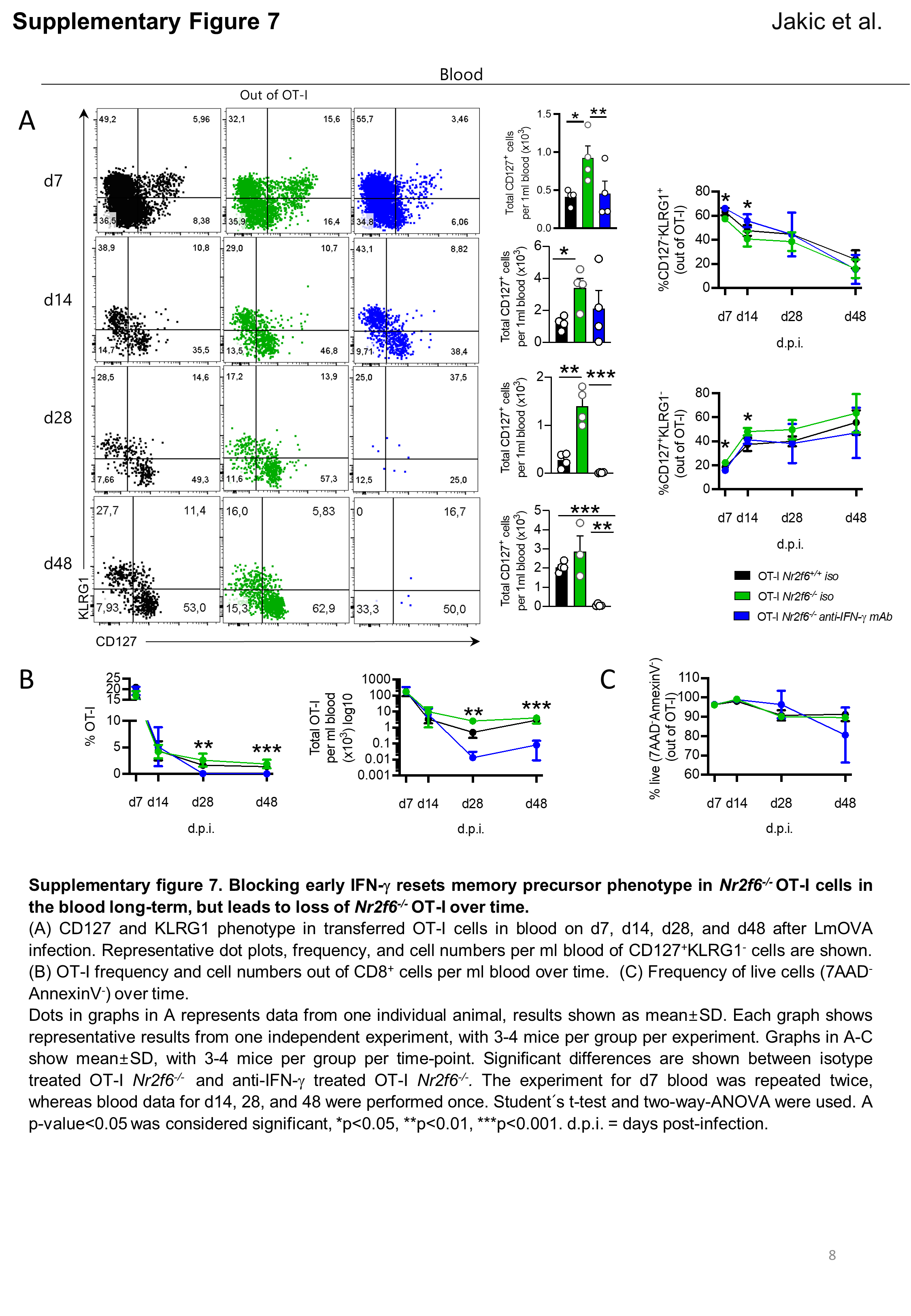

Supplement: Supplementary file 8 — Supplemantal Material Fig7 [file 41419_2021_3470_MOESM8_ESM.tif]
